# A RanBP2-type zinc finger protein functions in intron splicing in Arabidopsis mitochondria and is involved in the biogenesis of respiratory complex I

**DOI:** 10.1093/nar/gkab066

**Published:** 2021-02-28

**Authors:** Stéphane Bentolila, Andrew B Gipson, Alexander J Kehl, Lauren N Hamm, Michael L Hayes, R Michael Mulligan, Maureen R Hanson

**Affiliations:** Department of Molecular Biology and Genetics, Cornell University, Ithaca, NY 14853, USA; Department of Molecular Biology and Genetics, Cornell University, Ithaca, NY 14853, USA; Department of Molecular Biology and Genetics, Cornell University, Ithaca, NY 14853, USA; Department of Molecular Biology and Genetics, Cornell University, Ithaca, NY 14853, USA; Department of Chemistry and Biochemistry, California State University Los Angeles, Los Angeles, CA 90032, USA; Department of Developmental and Cell Biology, University of California Irvine, Irvine, CA 90032, USA; Department of Molecular Biology and Genetics, Cornell University, Ithaca, NY 14853, USA

## Abstract

The RanBP2 zinc finger (Znf) domain is a prevalent domain that mediates protein interaction and RNA binding. In Arabidopsis, a clade of four RanBP2 Znf-containing proteins, named the Organelle Zinc (OZ) finger family, are known or predicted to be targeted to either the mitochondria or the plastids. Previously we reported that OZ1 is absolutely required for the editing of 14 sites in chloroplasts. We now have investigated the function of OZ2, whose null mutation is embryo lethal. We rescued the null mutant by expressing wild-type *OZ2* under the control of the seed-specific ABSCISIC ACID-INSENSITIVE3 (ABI3) promoter. Rescued mutant plants exhibit severely delayed development and a distinctive morphological phenotype. Genetic and biochemical analyses demonstrated that OZ2 promotes the splicing of transcripts of several mitochondrial *nad* genes and *rps3*. The splicing defect of *nad* transcripts results in the destabilization of complex I, which in turn affects the respiratory ability of *oz2* mutants, turning on the alternative respiratory pathway, and impacting the plant development. Protein-protein interaction assays demonstrated binding of OZ2 to several known mitochondrial splicing factors targeting the same splicing events. These findings extend the known functional repertoire of the RanBP2 zinc finger domain in nuclear splicing to include plant organelle splicing.

## INTRODUCTION

Plant mitochondria were once free-living organisms, related to contemporary alpha-protobacteria, that were engulfed about 2 billion years ago by the ancestor of eukaryotic cells. It is now accepted that endosymbiosis gave rise to mitochondria and, in plant cells, to the chloroplasts, whose ancestors were cyanobacteria (1). Both organelles have retained their own genomes; however, a gradual loss of genetic content has occurred with a massive transfer of genes from the mitochondria and the chloroplasts towards the nucleus (2).

Because of their prokaryotic origins, gene expression in both organelles has retained some bacterial-like traits, but gene transfer and co-evolution with the nuclear genome has resulted in overlay of eukaryotic features (3,4). The collection of genes still present in the mitochondrial genome is comparable, though not identical, across plant, fungal and animal lineages, usually incorporating some components of the OXPHOS system, tRNAs and ribosomal RNAs and some proteins. The proper function of mitochondrial respiratory complexes and translation apparatus, due to the dual origin of some of their components, either mitochondrial- or nuclear-encoded, require a coordination of gene expression from these two physically different genomes. In addition to the organellar genes that have been transferred to the nuclear genome, many other nuclear-encoded proteins targeted to the organelles function in the control of organelle gene expression. The very complex organelle RNA metabolism, which encompasses several common post-transcriptional processes such as processing, editing and splicing, is a striking illustration of the increasing control of the nucleus over organelle gene expression. Many proteins involved in these processes are not derived from the endosymbiont but were acquired by the host-cell during the nuclear-organellar co-evolution.

Most of the introns found in the angiosperm mitochondrial genomes belong to the group II class, with the presence of a group I intron limited to the *cox1* gene, only in certain lineages (5). Mitochondrial introns are found in the coding sequences of protein coding genes and must be spliced out in order to produce functional products. Some of the introns are fragmented and need to be reassembled *in trans* before being removed. These trans-splicing events occur in the mitochondrially-encoded subunits of the NADH:ubiquinone oxidoreductase complex (complex I), *nad1*, *nad2* and *nad5*, and also in the *cox2* intron in *Allium cepa* (6–9). Since endosymbiosis, these group II introns have degenerated compared to their prokaryotic ancestors. In particular, most of the mitochondrial introns have lost their intron-encoded proteins or maturases; only one maturase gene, *matR*, has been retained in the fourth intron of *nad1* (10). As a result, splicing of these introns does not occur *in vivo* without the assistance of nuclear-encoded splicing factors (11).

Another type of RNA processing in chloroplasts and mitochondria that is facilitated by nuclear proteins is plant organelle RNA editing, which is a post-transcriptional event in which cytidines are converted to uridines at many sites in chloroplast and mitochondrial transcripts. We have identified OZ1 (Organelle Zinc finger 1), a protein containing a RanBP2-type zinc finger (Znf), as a chloroplast editing factor, raising the possibility that other OZ family members could be editing factors. Here, we investigate the function of OZ2 (At1g55040), which, when mutated, results in an embryo defective (EMB) phenotype, arresting development at the cotyledon stage (12,13). We rescued the homozygous *oz2* mutant embryo with a wild-type transgene driven by an embryo-specific promoter, a strategy developed to study post-embryonic functions of EMB genes (14). The *oz2* rescued mutant plants exhibit a marked delay in development with dark green and curled rosette leaves. These partially complemented mutants have a dramatic decrease in the splicing efficiency of six mitochondrial introns, five of which are located in *nad* transcripts, which encode subunits of Complex I. Our data show that the assembly of complex I is seriously compromised as a result of the defective splicing. Therefore, unexpectedly, OZ2 is not an editing factor, but a mitochondrial splicing factor whose knockout leads to an embryo-lethal phenotype. Thus, we have determined that a RanBP2 zinc finger-containing protein has a novel function as an essential splicing factor in higher plant mitochondria.

## MATERIALS AND METHODS

### Plant material

The Arabidopsis T-DNA insertion lines SALK_043472C and WiscDsLox233237_13N (CS849339) in the *OZ2* gene were ordered from the Arabidopsis Biological Resource Center (https://abrc.osu.edu/). After 3 days of stratification, seeds were planted in soil growing in a growth room (14 h of light/10 h of dark) at 26°C. Genotyping was done by polymerase chain reaction (PCR) with OneTaq Quick-Load 2X Master Mix (New England Biolabs) using primer pairs listed in Supplementary Table S1. The wild-type alleles were amplified using the primer pairs SALK_043472C-LP and SALK_043472C-RP, and CS84933-LP and CS84933-RP. The mutant alleles were amplified using the primer pairs SALK_043472C-RP and LBb1.3, and CS84933-RP and p745. The PCR products were sequenced at Cornell University Life Sciences Core Laboratories Center.

### Constructs used in this study

A 2411-bp fragment of the ABI3 (AT3G24650) promoter was amplified using a HindIII restriction site forward primer 5′-AAGCTTCAACAAACGACTAGTACTGATATATACATC and a NheI restriction site reverse primer 5′-GCTAGCCGTTGAAGTGGAAATGAAACAATAAACTAG, and the Platinum SuperFi Green PCR Master Mix (Invitrogen) and cloned into the pCR8/GW/TOPO vector (Invitrogen). The sequence of the cloned ABI3 promoter was verified by using the primers listed in Supplementary Table S1 and digested with HindIII and NheI. The insert was separated from the vector by electrophoresis, gel purified (PureLink Quick Gel Extraction Kit, Invitrogen) and ligated to a Gateway-converted pBI121 binary vector previously digested with HindIII and XbaI. The ligated product resulted in swapping the CaMV35S promoter for the ABI3 promoter in the Gateway pBI121 vector. The Gateway pBI121 vector had been engineered previously by changing the resistance marker from kanamycin to Basta resistance using a strategy similar to exchanging the promoter. The Basta resistance cassette was amplified from the pEarleygate 205 vector (15) using a *PmeI* restriction site forward primer 5′-GTTTAAACTTCTGAGATTTTTCAAATCAGTGC and a HindIII restriction site reverse primer 5′-AAGCTTTCGGATCTGATAATTTATTTGAAAATT. The amplified Basta cassette was cloned into the pCR8/GW/TOPO vector, digested with HindIII and PmeI, gel purified and ligated to a Gateway pBI121 vector previously digested with HindIII and PmeI to release the Kanamycin resistance cassette. The final binary vector pBI121-ABI3-GW-Basta is a Gateway-compatible binary vector whose transgene is under the control of the ABI3 promoter, and the selection marker is resistance to Basta.

The cDNA clone of OZ2 used in this study was reverse-transcribed by SuperScript^®^ III Reverse Transcriptase (Invitrogen) with the OZ2-R1 primer (Supplementary Table S1) from RNA extracted from wild-type Arabidopsis Columbia using PureLink^®^ RNA Mini Kit (Invitrogen). The full length OZ2 CDS was amplified with OZ2-F1 and OZ2-R1 primers and cloned into the pCR8/GW/TOPO vector; after verifying the sequence with primers listed in Supplementary Table S1, the OZ2 CDS was transferred to the pBI121-ABI3-GW-Basta by LR Clonase II (Invitrogen).

A recombinant protein that included the two RanBP2 Zn finger domains (residues 343P to 454R of the OZ2 protein) was expressed in pET28a (Novagen). An amplicon was produced from OZ2 cDNA with oligonucleotide primers (AtOZ2_ZnFing_For; AtOZ2_ZnFing_Rev, Supplementary Table S1) and cloned into the EcoRI and HindIII sites of the pET28a plasmid.

### Generation of transgenic plants

ABI3-*OZ2* (hereafter OZ*) in the pBI121 vector was transformed into *Agrobacterium tumefaciens* GV3101, and floral dip transformation of heterozygous *oz2/OZ2* plants (SALK_043472C) was performed as described in (16). Progeny were selected on soil by spraying Basta on young seedlings; resistant plants were genotyped to determine whether they were homozygous wild-type OZ2/OZ2/OZ*, heterozygous oz2/OZ2/OZ*, or homozygous mutant oz2/oz2/OZ*. The wild-type plants were discarded, the homozygous mutant plant was investigated, and the heterozygous transgenic plants were grown to the next generation. T_1_ progeny from the heterozygous T_0_ parental plants were also subjected to Basta selection, and several homozygous oz2/oz2/OZ* plants were recovered in the T1 generation.

### OZ2 localization

*OZ2* was amplified from *OZ2* cDNA with Phusion polymerase (Thermo Scientific) and the primers OZ2-F1 and OZ2-nostop-R1 using standard protocols. 3′-A overhangs were added with Taq (QIAGEN) by incubating at 37°C for 10 min. After purification, the amplicons were cloned into pCR8/GW/TOPO (Invitrogen) to use in a Gateway cloning reaction with a modified pEXSG vector (17) containing an EYFP C-terminal tag using LR Clonase II (Invitrogen) to produce pEXSG-OZ2-YFP.

Arabidopsis Col-0 plants and *Nicotiana benthamiana* were grown on soil in a long day (16 h) conditions for 3–5 weeks for Arabidopsis and 5–6 weeks for *N. benthamiana*. pEXSG-OZ2-YFP was transfected into Arabidopsis and *N. benthamiana* protoplasts using the method outlined in (18), using ∼3.0 × 10^5^ cells per transformation.

Protoplast mitochondria were stained with MitoTracker™ Orange CM-H_2_TMRos (500 nM; ThermoScientific), using DMSO as the solvent and W5 buffer. Protoplasts were incubated in the dark for 45 min and then were pelleted (1000 × g, 5 min) and resuspended in W5 buffer (500 μl). Protoplasts were imaged using a Zeiss Axio Observer LSM 710 microscope and C-Apochromat 40×/1.20 W Korr M27 objective.

### RNA analysis

Total RNA was extracted from Arabidopsis rosette leaves (4–5-week-old plants) by pulverizing green tissue with liquid nitrogen and using Trizol (Ambion) according to the manufacturer's instructions. RNA was further purified with the PureLink^®^ RNA Mini Kit (Invitrogen). The purified RNA was treated with Turbo DNase (Invitrogen). Mitochondrial RT-PCR products were amplified with the same set of primers used in (19). The real-time quantitative RT-PCR conditions and analysis are similar to the ones outlined in (20), except for the use of an updated CFX Maestro Software (Bio-Rad) that allows performance of an ANOVA. The primers used for the quantitative RT-PCR to measure the expression of mitochondrial transcripts are described in (21), except for nad2-ex2ex3 and nad2-ex2in2, which are given in Supplementary Table S1. The qRT-PCR primers to measure the expression levels of alternative respiratory pathway genes are described in (22) for AOX1a, NDA1, NDA2, NDB1, NDB2, NDB3, NDB4 and NDC1. The qRT-PCR primers for AOX1c, and AOX1d are described in (23) while the primers for AOX1b, and AOX2 were retrieved from qPrimerDB, a qPCR primer database (24). The poisoned primer extension assays were performed as described previously (25) with primers listed in Supplementary Table S1.

### Blue native gel and respiratory complex in-gel activity assay

Some of the rescued homozygous *oz2* mutants (oz2/oz2/OZ*) were able to produce a reasonable amount of viable seeds. Mitochondria were extracted from liquid-grown Arabidopsis seedlings following the protocol of Murcha and Whelan (26). The yield of mitochondrial extraction was evaluated by Bradford assay (Bio-Rad). The sample preparation for the Blue native (BN) gel was done according to the protocol of Schertl and Braun (27). The gel used for the BN-PAGE was a pre-cast Native PAGE Novex 3–12% Bis–Tris gel (Invitrogen). The conditions for the run were: 4°C, 150 V for 60 min, 250 V for 180 min. Approximately 50 μg of mitochondrial protein was loaded per lane. After completion of the run, slices of the gel were either stained with Coomassie R-250 according to the manufacturer's instructions, or incubated overnight with freshly prepared solutions for in gel-activity at room temperature following the protocol of Schertl and Braun (27). The reactions were then stopped by transferring the gels into fixing solution.

### Zinc binding analysis

Recombinant OZ2 protein containing the two RanBP2 Zn finger domains was expressed in *E. coli* cells (Rosetta 2 (BL21) DE3 pLYS) in 1 l cultures at 18°C using 1 mM of IPTG and purified on Ni-NTA resin. Recombinant protein was digested overnight with thrombin (1 U/mg protein) at 4°C, and dialyzed twice in 20 mM ammonium acetate. The mass spectrometry analysis of the recombinant OZ2 containing the two RanBP2 Zn finger domains was performed as in (28) with a Waters QTOF2 instrument with an electrospray ion voltage of 3.6 kV, a cone voltage of 40 V, and a desolvation temperature of 120°C. In addition to native conditions performed in 20% ammonium acetate proteins were denatured using 50% acetonitrile 0.1% formic acid to release bound zinc ligands.

### Yeast two-hybrid assay

The mature coding sequences (without predicted N-terminal mitochondrial transit peptide) of OZ2 and mitochondrial splicing factors ABO5, BIR6, MAT2, mCSF1, MISF26, MISF74, MTL1, OTP439, PMH2, RUG3, SLO3 and WTF9 were amplified using Y2H primer pairs listed in Supplementary Table S1. PCR products were first cloned into pCR8/GW/TOPO and then pGADT7GW and pGBKT7GW yeast two-hybrid destination vectors via Gateway cloning. Empty pGADT7GW and pGBKT7GW vectors were used as negative controls in yeast two-hybrid assays. Yeast mating strains PJ69-4a and PJ69-4α were individually transformed with pGADT7GW and pGBKT7GW plasmids, respectively. Single transformants were mated to produce diploid double-transformant yeast on YPAD agar plates. Yeast harboring testing pairs were grown in leucine- and tryptophan-deficient media overnight; then, 10 μl of each culture was spotted onto leucine-, tryptophan-, histidine-, adenine-deficient media plates after being diluted with water to OD600 0.5, 0.05 and 0.005. Survival/growth plates were imaged after three days of incubation at 30°C.

### Bimolecular fluorescence complementation (BiFC)

The full-length coding sequences (including N-terminal mitochondrial transit peptides but without the stop codon) of OZ2 and mitochondrial splicing factors ABO5, BIR6, MAT2, mCSF1, MISF26, MISF74, MTL1, OTP439, PMH2, RUG3, SLO3 and WTF9 were amplified using BiFC primer pairs listed in Supplementary Table S1. PCR products were first cloned into pCR8/GW/TOPO and then pEXSG-nYFP and pEXSG-cYFP BiFC destination vectors via Gateway cloning. Strain *A. tumefaciens* GV3101::pMP90RK were transformed via electroporation using 1 μg plasmid. Electroporation was conducted with the following parameters: capacitance 25 μF, voltage 2.0 kV, resistance 200 Ω, pulse length ∼5 ms. Agrobacteria were selected on LB agar plates containing kanamycin (50 μg/ml), gentamicin (25 μg/ml) and carbenicillin (50 μg/ml).

5-ml cultures of individual transformed Agrobacteria were incubated for 2 days at 28°C and resuspended in a solution of 2-(*N*-morpholine)-ethanesulphonic acid (pH 5.6), 20 mM Na_3_PO_4_, and 150 mM acetosyringone. Infiltration samples were made by mixing bacterial cultures carrying pEXSG-nYFP, pEXSG-cYFP and P19 at equal OD_600_ to a final OD of 0.9. Leaves of 4- to 6-week-old *N. benthamiana* plants grown in long-days conditions were agroinfiltrated as described in (29). 2–3 days post-infiltration, 2-mm squares were cut from the infiltrated leaf area and imaged using fluorescence microscopy as described above.

## RESULTS

### Arabidopsis *oz2* mutant plants are embryo-lethal but can be rescued by the use of the ABI3 promoter

**Figure 1.**
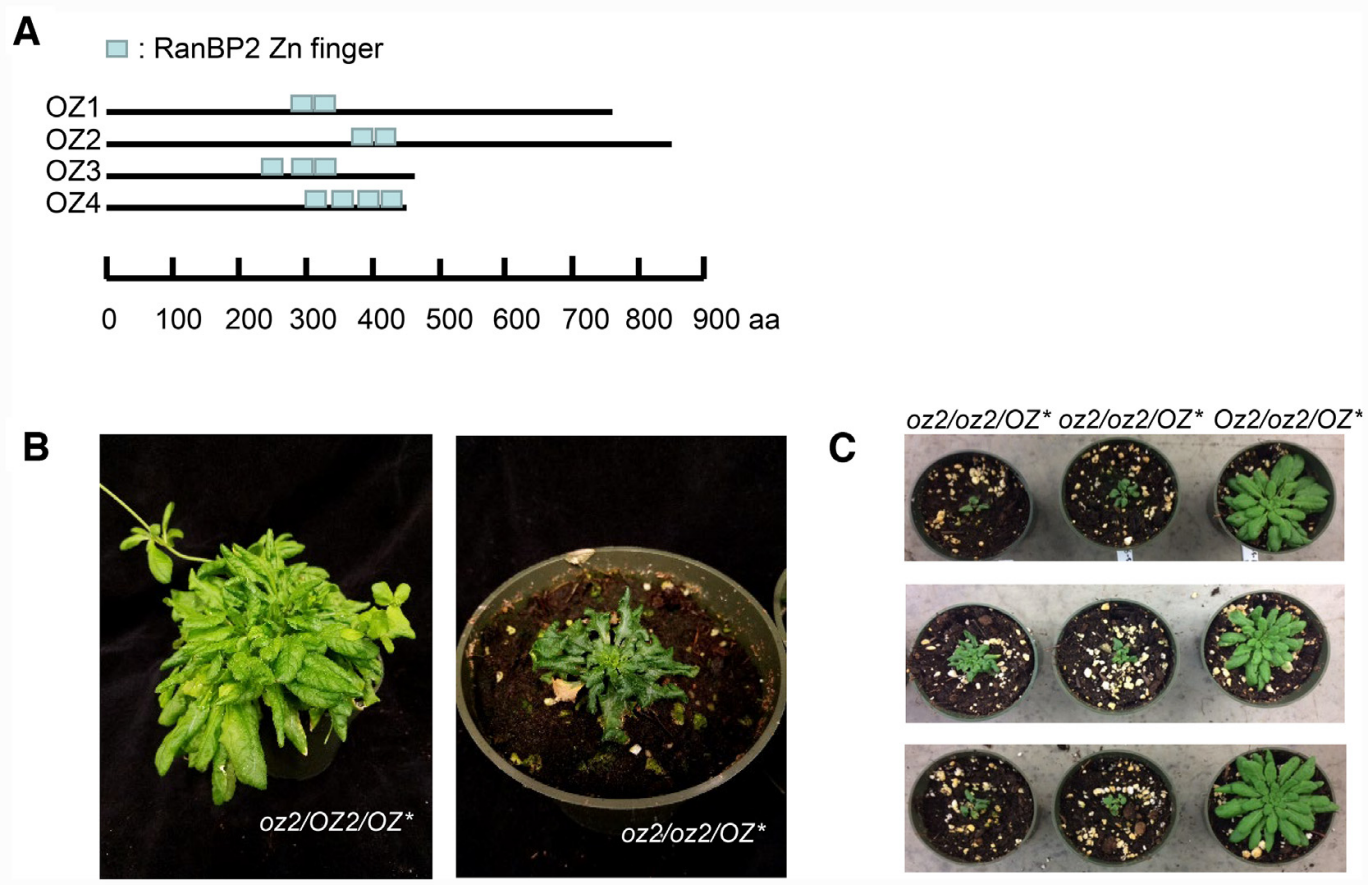
OZ2 belongs to a four-member family, and its partial complementation in a mutant background leads to a stunted growth phenotype. (**A**) Schematic representation of each OZ protein with the location of their RanBP2-type zinc finger motifs shown in blue. (**B**) The rescued oz2/oz2/OZ2* mutant plant exhibits slow growth and dark curled leaves. (**C**) Identification of several independent T1 oz2/oz2/OZ2* plants confirm the morphological defect caused by the *oz2* mutation. Six T1 oz2/oz2/OZ2* plants show a stunted phenotype similar to one of the T_0_ plants previously identified. In each panel, the three T1 plants are progeny from a T_0_Oz2*/oz2/OZ2 plant. The three panels represent three different transgenic events.

Both OZ1 and OZ2 contain two Znf domains while OZ3 and OZ4 have three and four Znf domains, respectively (Figure 1A). The RanBP2 Znf domain is 30 aa long and is a C4-type member of the broader zinc finger family, known to have unique functions and an unusually diverse distribution in plants (30).

In order to investigate the function of OZ2, we obtained two independent insertional mutant lines from the Arabidopsis Biological Resource Center (https://abrc.osu.edu/) (Supplementary Figure S1A). Sequencing of the mutant allele confirmed the location of the T-DNA insertion in the third exon for SALK_043472C, while the insertion in WiscDsLox233237_13N was found to be in the fourth intron instead of the fourth exon as annotated in the database. No homozygous mutant plants were found in the segregating progeny of a heterozygous parent plant of either insertional line, indicating that the *oz2* mutation is embryo lethal (Supplementary Figure S1B).

To overcome the embryo lethality of the *oz2* mutation and study the function of OZ2, we attempted to rescue the mutant embryo by complementing heterozygous lines (*oz2/OZ2*) with a cDNA carrying the wild-type coding sequence under the control of the seed-specific ABSCISIC ACID-INSENSITIVE3 (ABI3) promoter: ABI3-OZ2 (OZ*) (14). The ABI3 promoter drives the expression of the *OZ2* wild-type transgene during embryogenesis, allowing the development of homozygous *oz2* mutant embryos, but leading to the appearance of mutant phenotypes in later stages of development when it is down-regulated. We were able to retrieve one rescued homozygous *oz2* mutant seedling (oz2/oz2/OZ*) in the T_0_ population. This mutant plant shows a distinctive morphological phenotype when compared to the wild-type plant, with delayed growth and dark green, curled leaves (Figure 1B). Eleven heterozygous (*oz2*/OZ2/OZ*) T_0_ plants carrying the ABI3-*OZ2* transgene were also identified (Figure 1B). Segregating progeny from some of these heterozygous plants showed the same morphological phenotype as the T_0_ homozygous *oz2* mutant plant (oz2/oz2/OZ*), demonstrating that the *oz2* mutation causes this defective phenotype (Figure 1C).

**Figure 2.**
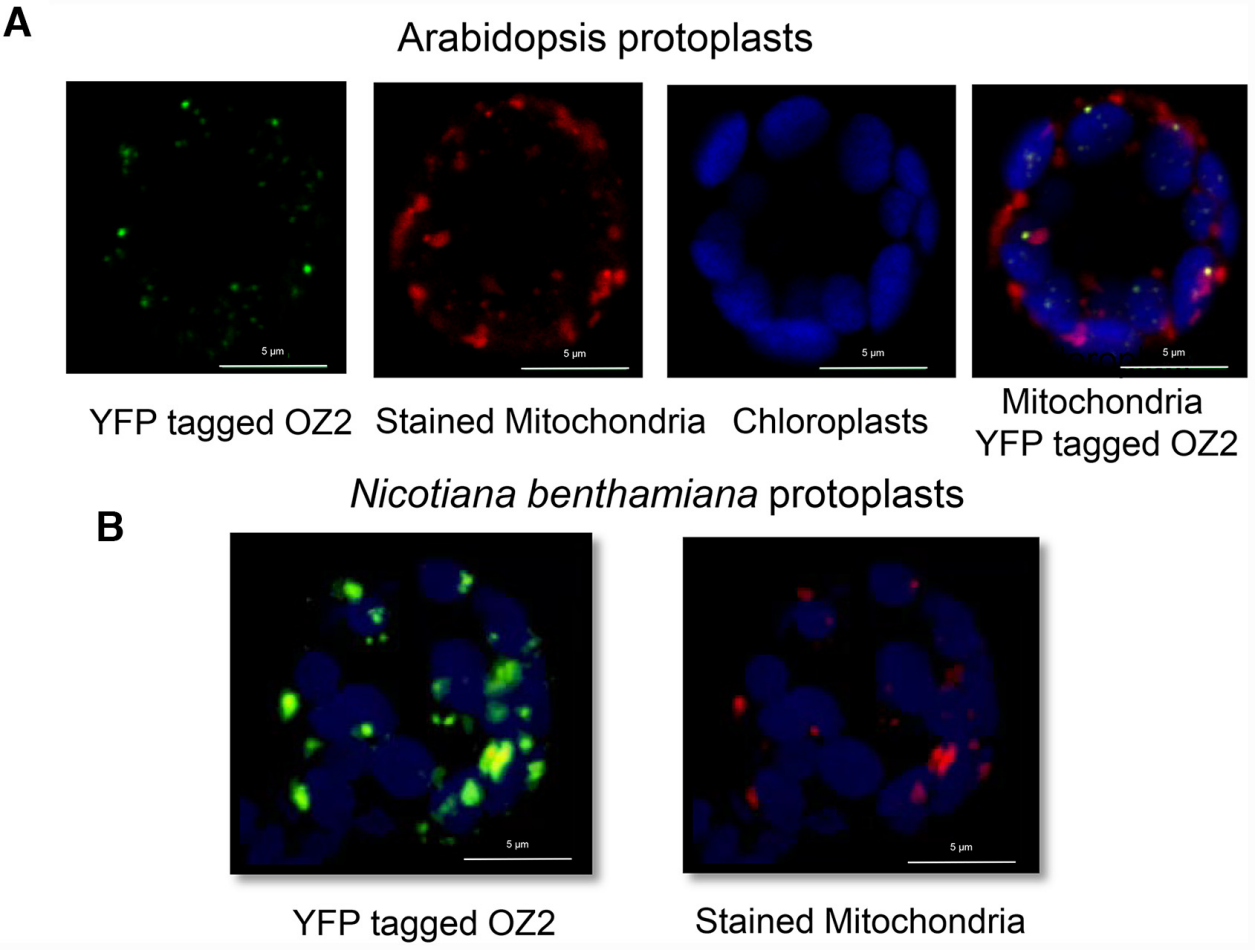
OZ2 is targeted to mitochondria. (**A**) Transfection of Arabidopsis protoplasts with OZ2 fused to YFP. From left panel to right panel: OZ2 location appears as green dots, mitochondria are stained in red with MitoTracker, chloroplasts autofluorescence is artificially colored blue, overlay of these three panels demonstrate the location of OZ2 in mitochondria as yellow (green + red) dots. (**B**) Transfection of *Nicotiana benthamiana* protoplasts with OZ2 fused to YFP. Some of the green spots (OZ2-YFP) can be seen to co-localize with the red spots (mitochondria).

The Arabidopsis subcellular database SUBA (http://suba.live/) strongly supports a plastid location for OZ2; however, the most recent version of TargetP (TargetP-2.0), a predictive software for the subcellular localization of proteins, predicts the localization of OZ2 to be mitochondrial with high confidence (31). Predotar, another predictive software, favours a plastidial over a mitochondrial localization of OZ2, but only by a slight margin (32). To resolve these ambiguities and find out the subcellular localization of OZ2, we transiently expressed in Arabidopsis or *N. benthamiana* protoplasts a YFP translational fusion comprising the complete coding sequence of OZ2 (Figure 2). In protoplasts of both species, confocal microscopy demonstrated that the YFP fluorescence co-localizes with the fluorescent signal from Mitotracker Orange that specifically labels mitochondria. Thus, this analysis demonstrated that OZ2 was targeted to mitochondria and not plastids, supporting a previous localization study in Arabidopsis (12)

### Splicing of most transcripts of the complex I and *rps3* is severely impaired in *oz2* mutants

Since OZ1 is an essential plastid editing factor, and OZ2 is a mitochondrial-targeted protein closely related to OZ1, we tested whether a defect in mitochondrial editing could be the source of the morphological phenotype caused by the *oz2* mutation. Survey of the mitochondrial editome by bulk-sequencing of the RT-PCR products corresponding to the whole set of mitochondrial genes did not reveal any defects in editing in the *oz2* mutant T_0_ plant (oz2/oz2/OZ*) (Supplementary Figure S2A). However, during the amplification of the RT-PCR products, four mitochondrial genes, *nad2*, *nad5*, *nad7* and *rps3*, exhibited a ladder pattern with a marked reduction of the mature product in the *oz2* mutant T_0_ plant (Supplementary Figure S2B). A feature common to these four genes is that splicing is involved in their transcript maturation. In addition, even though the RT-PCR reaction was not meant to be quantitative, there was also a noticeable reduction of the amplified cDNA for two other *nad* genes, *nad1* and *nad4* in the *oz2* mutant (Supplementary Figure S2C).

We performed quantitative RT-PCR (qRT-PCR) assays for all the mitochondrial transcripts using a set of primers that allowed survey of not only the abundance of the transcripts, but also the efficiency of splicing by discriminating between the spliced and unspliced transcripts (21). This analysis was conducted on three wild-type T_1_ plants and four *oz2* mutant (oz2/oz2/OZ*) plants: the T_0_ oz2/oz2/OZ* plant and three of the T_1_ plants derived from three different heterozygous T_0_ oz2/OZ2/OZ* plants (Figure 1B and C). Therefore, the four *oz2* mutant plants analysed were produced via four independent transgenic events, presumably with random insertion of the ABI3:*OZ2* construct in the genome.

**Figure 3.**
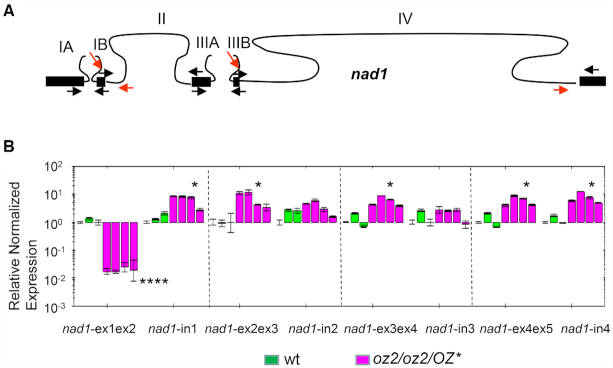
OZ2 is a mitochondrial splicing factor. (**A**) Gene model for *nad1*. Black boxes (lines) represent exons (introns). Maturation of *nad1* necessitates four splicing events, two *cis*-splicing events (II and IV), and two *trans*-splicing events (I and III). Arrows indicate the primers used in the qRT-PCR analysis; red arrows indicate the primers used to quantify the amount of unspliced transcripts. (**B**) qRT-PCR analysis of *nad1* transcript species demonstrated that the amount of spliced intron 1 transcript (*nad1*-ex1ex2) is severely reduced in oz2/oz2/OZ* (around 60 times), while the amount of unspliced precursor (*nad1*-in1) is slightly higher in oz2/oz2/OZ* than in the wild-type (wt) plants (around 5 times). Splicing of intron 1 is the only splicing defect for *nad1* as the other spliced transcripts, nad1-ex2ex3, nad1-ex3ex4, nad1-ex4ex5, exhibit a slight but significant increased amount in *oz2* compared to the wild-type plants. The assay was performed on the RNAs from plants shown in Figure 1: three wild-type T1 plants, three oz2/oz2/OZ2* T_1_ plants, and one oz2/oz2/OZ2* T_0_ plant. Three technical replicates were measured for each data point (*P< 0.05, **P< 0.01, ***P< 0.001, ****P< 0.0001).

The qRT-PCR analysis sheds light on the molecular defect caused by the *oz2* mutation that resulted in the reduction of mature products for some of the *nad* genes as observed after RT-PCR (Supplementary Figure S2B and C). The maturation of some mitochondrial *nad* genes is quite complex and requires both *cis-* and *trans*-splicing events (8,9,33). For instance, the *nad1* gene is split into five coding segments localized on three precursor transcripts that are transcribed from three genomic areas scattered over 136 kb in the Arabidopsis mitochondrial genome (Figure 3A). Two *trans*-splicing events and two *cis*-splicing events are necessary for the complete maturation of the *nad1* transcript. The qRT-PCR results revealed a major and highly significant (*P* < 0.0001) reduction of the intron 1 spliced transcript, denoted nad1-ex1ex2 in Figure 3B. Its transcript abundance is reduced around 60 times in the *oz2* mutant compared to the wild-type plant. The RNA comprising intron IB, one of the precursors of the intron 1 spliced product, exhibited a significant (*P* < 0.05) increase in the *oz2* mutant (Figure 3B). This increase is more moderate than the decrease of the intron 1 spliced transcript, 5 times versus 60 times, respectively, supporting a true defect in splicing instead of a destabilization of the precursor transcript. None of the other *nad1* spliced species, pertaining to the splicing of introns 2, 3 or 4, exhibited a reduced amount in the *oz2* mutant; the abundance of these spliced products was significantly (*P* < 0.05) but moderately (5–7 times) increased in the *oz2* mutant (Figure 3B). The specificity of the effect of *oz2* mutation, with a marked reduction of only the intron 1 spliced transcript, strongly suggests a defect in splicing rather than a global destabilization across the entire *nad1* transcript.

**Figure 4.**
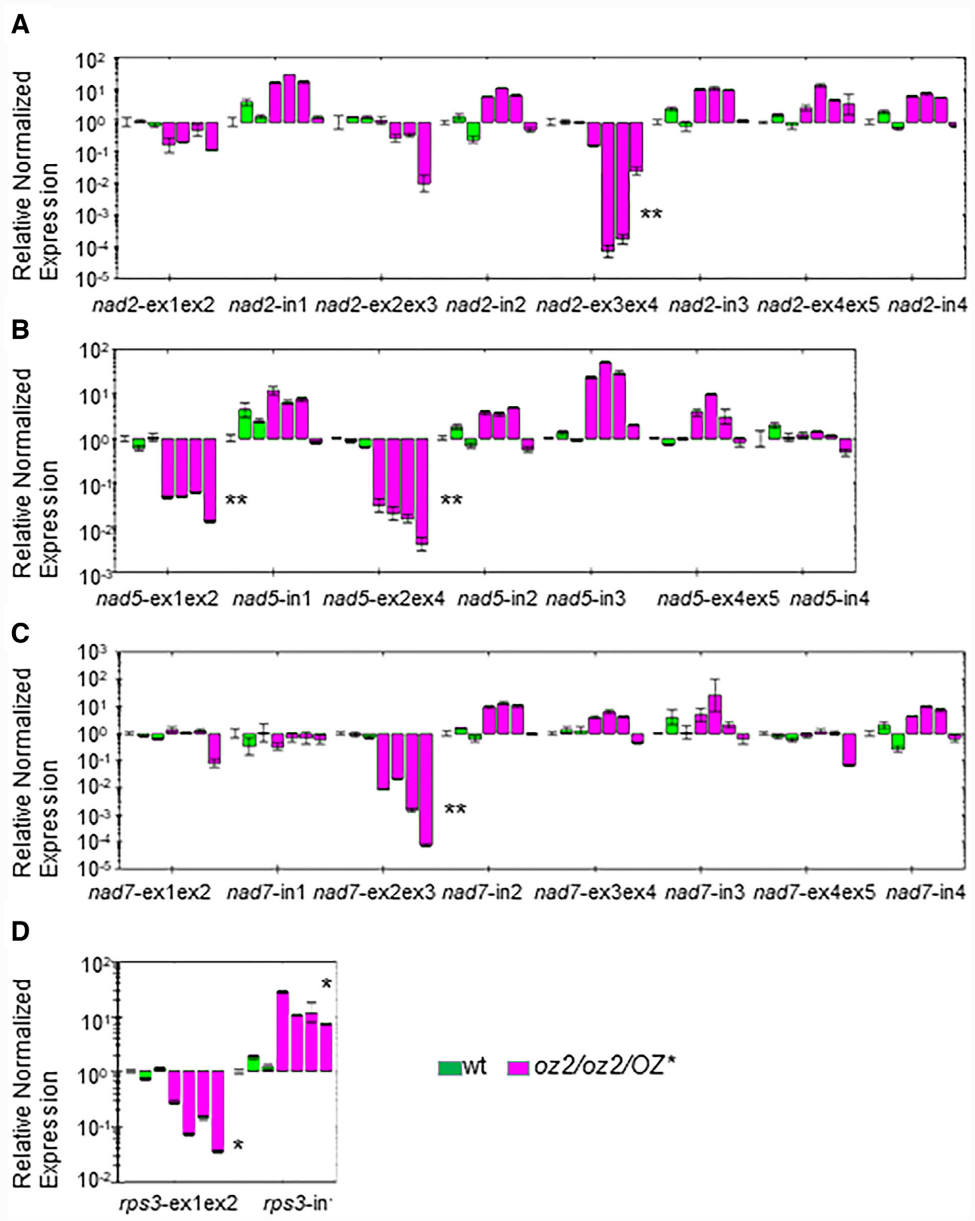
OZ2 is a mitochondrial splicing factor that controls the splicing events of complex I transcripts and *rps3*. (**A**) Survey of the splicing in *nad2* by qRT-PCR analysis. The spliced species without intron 3 shows a significant reduction in oz2/oz2/OZ*. (**B**) Survey of the splicing in *nad5* by qRT-PCR analysis. Two of the spliced species of this transcript show a significant reduction in the oz2/oz2/OZ* mutant. (**C**) Survey of the splicing in *nad7* by qRT-PCR analysis. The spliced species without intron 2 shows a significant reduction in oz2/oz2/OZ* (**D**) Survey of the splicing in *rps3* by qRT-PCR analysis. The spliced *rps3* shows a significant reduction in the oz2/oz2/OZ* mutant while the unspliced precursor amount is significantly increased. The order of the plants assayed is similar to Figure 3: three wild-type T1 plants, three oz2/oz2/OZ2* T_1_ plants, and one oz2/oz2/OZ2* T_0_ plant. Three technical replicates were measured for each data point (*P< 0.05, **P< 0.01)

Defective splicing events caused by the *oz2* mutation were observed in other *nad* transcripts. Like *nad1*, the *nad2* gene showed a very significant (*P*<0.01) reduction in abundance of only one of its spliced species (Figure 4A). The intron 3 spliced transcript is reduced 830 times in abundance on average in the *oz2* mutant plant relative to wild-type. None of the other transcript species assayed in *nad2* showed a significant variation in the mutant plant. The increase in the precursor to this spliced species, *nad2*-in3 in Figure 4A, although not significant, further supports the involvement of *OZ2* specifically in splicing, since the reduced amount of the spliced product can thus not be caused by a decreased availability of the precursor. Unlike the *nad1* first intron which is *trans*-spliced, the third intron of *nad2* is *cis*-spliced. Two of the *nad5* spliced products are very significantly (*P* < 0.01) reduced in abundance in the *oz2* mutant (Figure 4B). The intron 1 *cis*-spliced transcript reduction is about 25 times in the *oz2* mutant plant while the intron 3 or intron 4 *trans*-spliced transcript reduction amounts to 60 times. Due to the short length of *nad5* exon 3 (22 nt), we did not try to discriminate between a defective splicing of intron 3 or intron 4 by using a qRT-PCR assay. The splicing of intron 2 in the *nad7* transcript is the last example of a defective splicing event in the *nad* transcripts found in the *oz2* mutant plants (Figure 4C).

The only non-complex I gene whose transcripts exhibit defective splicing is *rps3*, encoding the ribosomal protein S3 (Figure 4D). The significant reduction of spliced *rps3* in *oz2* is moderate, reaching about a tenth of the amount of the wild-type plant, while the amount of unspliced transcript is about 10 times the amount found in the wild-type.

In summary, among 68 transcript species tested by qRT-PCR assays, six transcripts, all of them spliced products, showed a very significant reduction of their abundance in the *oz2* mutant plant versus the wild-type plant (Supplementary Figure S3). The decrease ranged from 0.11 (*rps3*) to 0.0011 (*nad7*-ex2ex3). Eleven transcripts exhibited a significantly increased abundance in the *oz2* mutant relative to the wild-type, ranging from 3 to 10 times. These transcripts could be spliced products, e.g. *ccmFc*-ex1ex2, unspliced products, e.g. *nad1*-in1ex2, or ones not requiring splicing for their maturation, e.g. *atp1* (Supplementary Figure S3).

In order to confirm the splicing defect caused by the *oz2* mutation, we performed a semi-quantitative RT-PCR assay comparing the amount of amplified products, spliced and unspliced, in the wild-type versus oz2 (oz2/oz2/OZ*) at different number of cycles (Supplementary Figure S4). At 30 cycles, a *rps3* spliced product is detectable in the wild-type lanes, but not in the *oz2* lanes (Supplementary Figure S4A). Conversely, an unspliced *rps3* product is detectable in the *oz2* lanes but not in the wild-type lanes. At 35 cycles, the *rps3* spliced product is now detectable in the *oz2* lanes; however, its abundance is reduced when compared to the wild-type lanes. The opposite is true for the unspliced product; its abundance is higher in the *oz2* lanes than in the wild-type lanes. The results of this assay recapitulate the data from the qRT-PCR assay with a roughly 10-time reduction of spliced (unspliced) transcript in the *oz2* (wild-type) plant. We also tested the abundance of the *nad2*-intron 3 spliced and unspliced product (Supplementary Figure S4B). At 35 cycles, the spliced product is readily detectable in the wild-type lanes but not in the *oz2* mutant lanes. The unspliced product is more abundant in the *oz2* lanes than in the wild-type lanes. At 40 cycles, there is still no detectable trace of spliced *nad2* in the *oz2* lanes, while the amount of amplified product is easy to detect in the wild-type lanes. These results validate the qRT-PCR data that estimated the spliced product to be on average 830 times less abundant in the oz2 mutants than in the wild-type plants.

**Figure 5.**
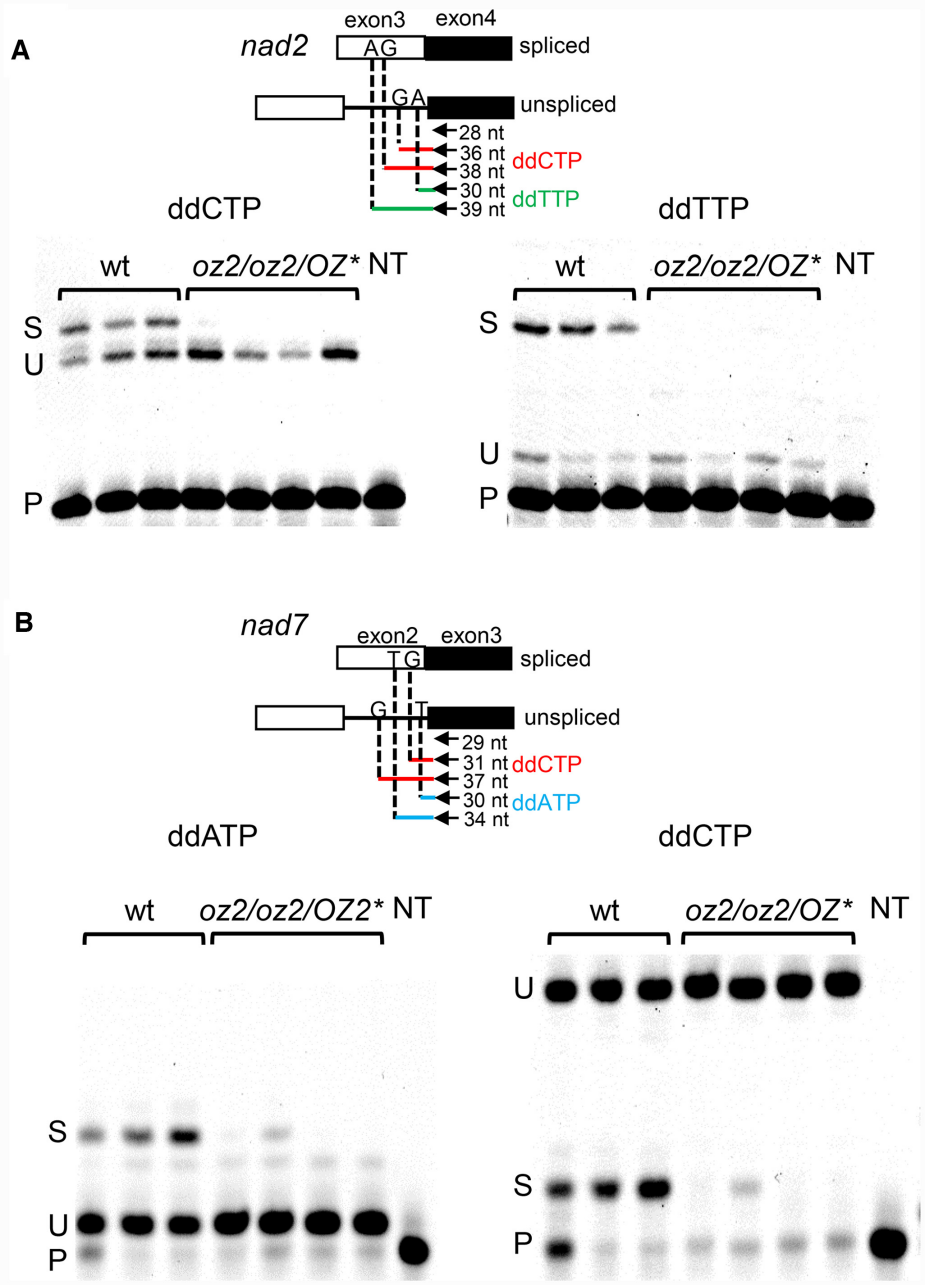
Poisoned primer extension (PPE) assay confirms that *oz2* mutation results in a splicing defect. Above the PPE gels is a schematic representation of the two cDNAs, spliced or unspliced, that were used as templates for the PPE reaction. The position of the primer represented by an arrow is shown upstream of the junction of exon (square) with intron (line). The length of the PPE products is indicated for each chain terminator used and each extension product is color coded (red for ddCTP, green for ddTTP, and blue for ddATP). The first nucleotide complementary to the dideoxynucleotide used in the PPE reaction that will stop the extension is indicated on either the exon (spliced) or the intron (unspliced). (**A**) *nad2* PPE assay shows a detectable amount of spliced product in the wild-type lanes, while there is no detectable spliced product in the oz2/oz2/OZ2* lanes. Conversely, some unspliced product is detectable in both the wild-type and the oz2/oz2/OZ2* lanes. Left panel: the PPE reaction included the chain terminator ddCTP such that the extension terminated after the addition of 8 or 10 nucleotides on unspliced or spliced templates, respectively. Right panel: the PPE reaction included the chain terminator ddTTP such that the extension terminated after the addition of 2 or 11 nucleotides on unspliced or spliced templates, respectively. (**B**) *nad7* PPE assay shows a detectable amount of spliced product in all the wild-type lanes, while there is detectable spliced product in only one of the oz2/oz2/OZ2* lanes. Conversely, some unspliced product is detectable in both the wild-type and the oz2/oz2/OZ2* lanes. Left panel: the PPE reaction included the chain terminator ddATP such that the extension terminated after the addition of one or five nucleotides on unspliced or spliced templates, respectively. Right panel: the PPE reaction included the chain terminator ddCTP such that the extension terminated after the addition of two or eight nucleotides on spliced or unspliced templates, respectively. P: primer, U: unspliced, S: spliced, wt: wild-type plant, *oz2*: *oz2* mutant plant, NT: no template.

A poisoned primer extension (PPE) assay was also conducted to confirm the splicing defect for two of the affected transcripts in the *oz2* mutant plants (Figure 5). In this assay, a fluorescent primer is extended in the presence of a template, either RNA or cDNA, and a mix of deoxyribonucleotide triphosphates and one dideoxynucleotide triphosphate. The primer is located on the exon just upstream of the junction intron/exon to be tested. The extension reaction will stop at the first nucleotide in the template that is complementary to the dideoxynucleotide in the reaction mix. The dideoxynucleotide is chosen so that the length of the extension product differs between the spliced versus unspliced template. Assaying the splicing of the third exon in the *nad2* transcript by a PPE reaction shows both spliced and unspliced extension products for the wild-type lanes but no detectable spliced product in the *oz2* lanes (Figure 5A). We performed this PPE assay with two different dideoxynucleotides, either ddCTP or dTTP, and in both experiments there was no detectable spliced product in the *oz2* lanes. We also assayed the splicing of *nad7*-intron 2 by PPE with two different dideoxynucleotides, either ddATP or ddCTP (Figure 5B). Like for *nad2*, both spliced and unspliced products are present in wild-type lanes, while the spliced product is absent in three of the *oz2* mutant lanes. A faint band representing the spliced product is detectable in one of the *oz2* lanes, but with much less intensity than in the wild-type lanes.

In conclusion, our data strongly support a role of OZ2 in the splicing of most mitochondrial complex I and *rps3* transcripts. The splicing defect affects one to two splicing events per transcript and can alter both *trans*-splicing (*nad1*-intron 1, *nad5*-intron 2 or intron 3) and *cis*-splicing processes (*nad2*-intron 3, *nad5*-intron 1, *nad7*-intron 2 and *rps3*).

### *oz2* mutants are severely impaired in respiratory complex I

**Figure 6.**
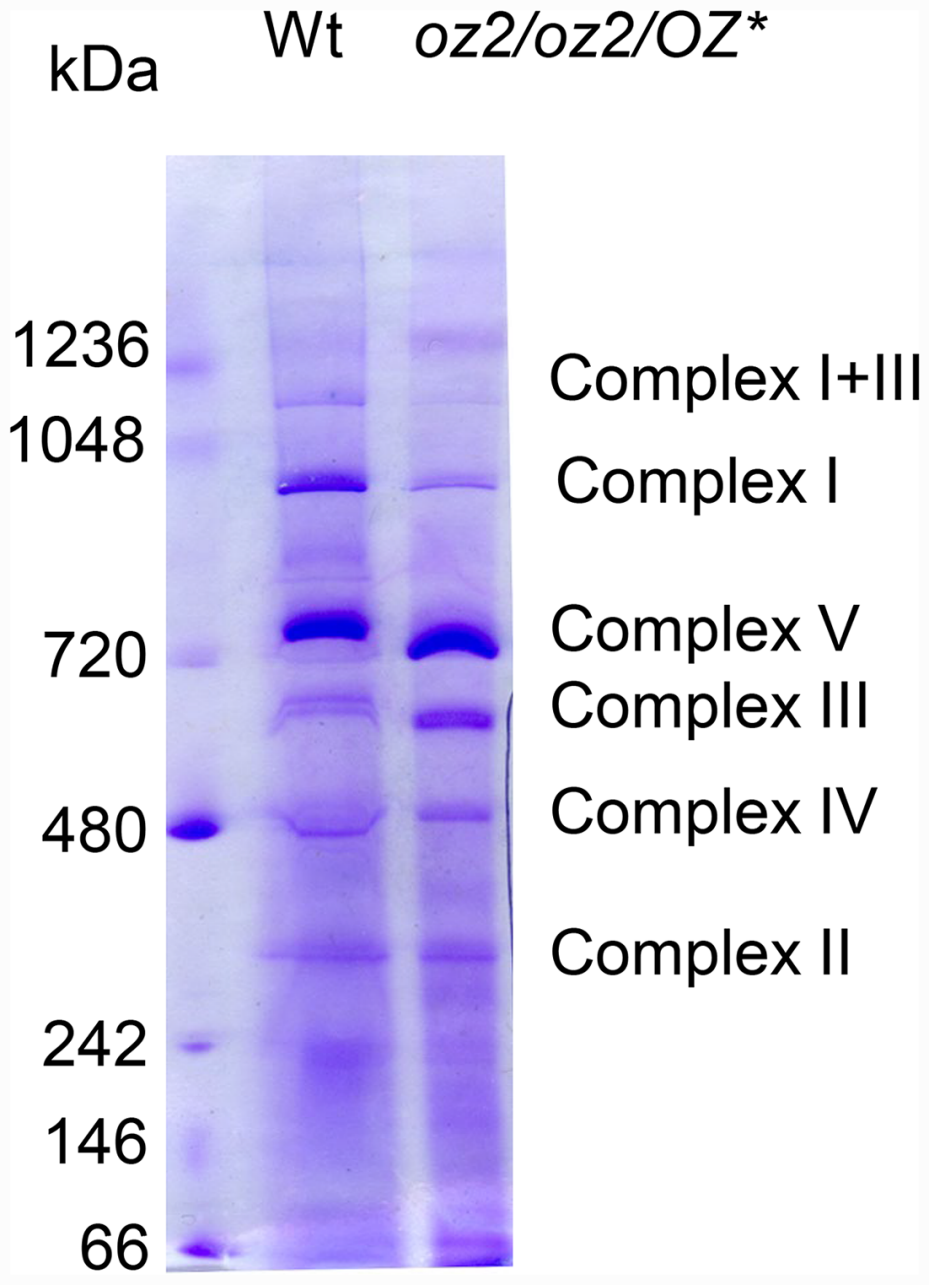
Blue native gels demonstrate that only complex I is seriously impaired in the oz2/oz2/OZ2* mutant. Arabidopsis mitochondrial proteins were solubilized by digitonin and subsequently separated by 1D BN-PAGE. After electrophoresis, slices of the gel were stained with Coomassie.

Given the molecular defects caused by the *oz2* mutation, we considered that a respiratory dysfunction, more specifically a deficiency in complex I, could be the source of the developmental alterations observed in the *oz2* mutant plants. Most of the NAD proteins are highly hydrophobic and therefore lack effective antibodies. We examined the steady-state levels of the different respiratory chain complexes after separation by blue-native PAGE (BN-PAGE) (Figure 6). An approximately equivalent amount of mitochondrial protein (ca. 50 μg/lane) from both the wild-type and the *oz2* mutant plant (oz2/oz2/OZ*) was loaded onto the gel. Coomassie staining of the gel after BN-PAGE shows that most of the bands representing the different complexes are present in the same amount in both the wild-type and the *oz2* lanes, except for complex I (Figure 6). A marked reduction of complex I bands, representing both complex I and supercomplex I + III, is apparent in the *oz2* lane compared to the wild-type lane. An in-gel NADH dehydrogenase activity assay confirmed the significantly decreased amount of complex I observed in the Coomassie-stained gel (Supplementary Figure S5). Succinate dehydrogenase (complex II), cytochrome *c* oxidase (complex IV), and ATPase (complex V) were also tested and showed similar activities in *oz2* and wild-type plants, confirming the observations from the Coomassie stained gel (Supplementary Figure S5)

**Figure 7.**
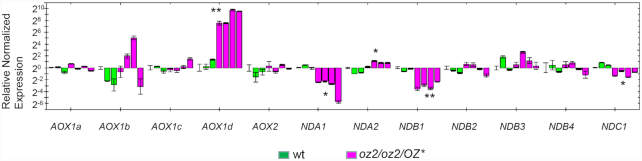
Quantitative RT-PCR analysis of alternative transport pathway transcripts show a strong induction of AOX1d in the oz2/oz2/OZ2* mutants. The assay was performed on the same RNAs from the same plants tested for the whole mitochondrial transcriptome: three wild-type T_1_ plants, three oz2/oz2/OZ2* T_1_ plants, and one oz2/oz2/OZ2* T_0_ plant. Three technical replicates were measured for each data point (*P< 0.05, **P< 0.01).

Previously described Arabidopsis mutants lacking complex I have elevated levels of genes involved in the alternative respiratory pathway (22,34–36). We assessed the level of expression of alternative oxidase (*AOX*) genes, isoforms *AOX1a-d* and *AOX2*, and the alternative external (*NDB1–4*) and internal (*NDA1–2* and *NDAC1*) *NAD(P)H* dehydrogenase genes in the *oz2* mutant by qRT-PCR analysis (Figure 7). This assay revealed a significant (*P* < 0.01) and very high induction of the expression of *AOX1d* in the *oz2* mutant plants, whose steady state level was ∼270-fold higher than in the wild-type plants. The *NDA2* gene also exhibited a significant (*P* < 0.05) but moderate induction of its expression in the *oz2* mutant plants, with a steady state level ∼3-fold higher than in the wild-type plants. Three alternative *NAD(P)H* dehydrogenase genes, *NDA1*, *NDB1* and *NDC1*, show a significant reduction of their expression in the *oz2* mutants; however, the magnitude of this decrease (3–11-fold) is much less pronounced than the induction of *AOX1d* (Figure 7).

Altogether these results lead us to conclude that *oz2* mutants are complex I-deficient plants. The splicing defect of *nad* transcripts results in the destabilization of complex I, which in turn affects the respiratory ability of *oz2* mutants, turning on the alternative respiratory pathway, and impacting the plant development with stunted growth and curly leaves. These developmental abnormalities are typical of complex I mutants, e.g. (22,35,37).

### OZ2 binds two zinc ions

**Figure 8.**
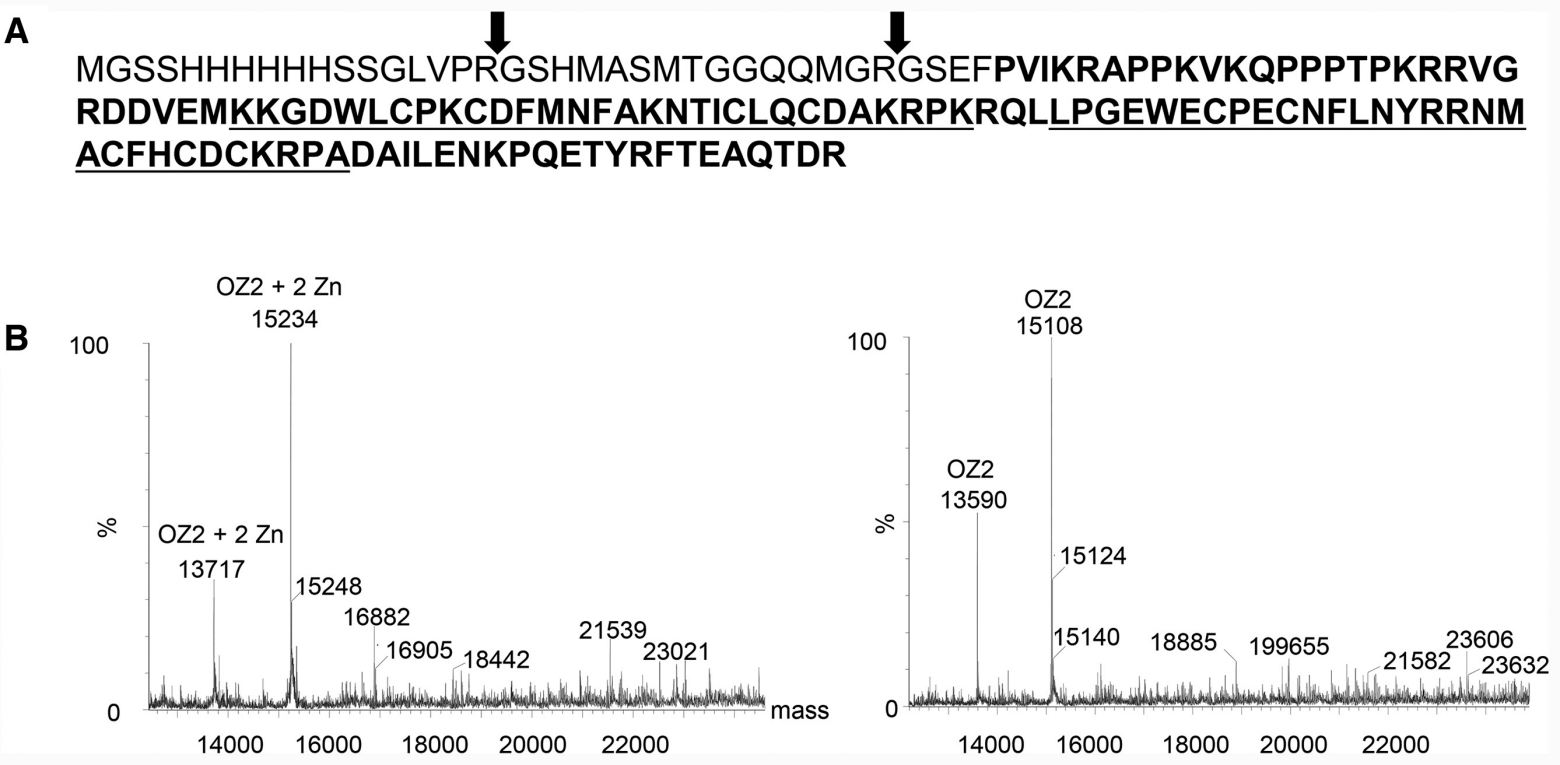
OZ2 binds two zinc atoms per polypeptide. (**A**) Amino acid sequence of the recombinant protein subjected to mass spectrometry analysis. Bolded sequences represent part of the recombinant OZ2 protein (aa 343–454), underlined are the two RanBP2Zn finger domains. Thrombin cleavages confirmed by MS are indicated by arrows. (**B**) Left, MS spectra indicate the projected mass for OZ2 under native conditions. At right, spectra are for denaturing conditions. The mass difference between the native and denatured conditions (126–127 Da) corresponds to the mass of two zinc atoms.

The structure of the RanBP2 Zn finger domain was solved with the first domain of the ZNF265 (also called ZRANB2 and Zis), a human splicing factor (38). NMR spectra in both the absence and presence of Zn showed a substantial increase in chemical shift dispersion in the presence of Zn, demonstrating this domain to be a genuine zinc-binding domain. We expressed 112 aa of OZ2 encompassing the two RanBP2 Znf domains in *E. coli* and performed mass spectrometry (MS) analysis of this polypeptide under native and denaturing conditions (Figure 8). Native MS can determine the molecular mass of polypeptides under conditions that retain prosthetic groups so that covalently bound ligands can be identified based on the difference between native and denaturing MS spectra. Recombinant OZ2 protein showed two peaks under native conditions with masses of 15 234 and 13 717 (Figure 8B). The presence of two polypeptides after digestion with thrombin is explained by the presence of two cleavage sites, one canonical (LVPRGS) and one cryptic (QMGRGS) (Figure 8A). Under denaturing conditions, the masses of these two polypeptides are 15 108 and 13 590, respectively. The difference in mass between these two polypeptides, 126–127 Da, corresponds to two zinc atoms. Although zinc has an atomic mass of 65.4, cysteines coordinate zinc directly to the sulfur atom with loss of a proton. Therefore, the observed mass difference is consistent with two bound zinc atoms, likely one zinc atom per RanBP2 Zn finger domain.

### OZ2 binds specifically to some known splicing factors

**Figure 9.**
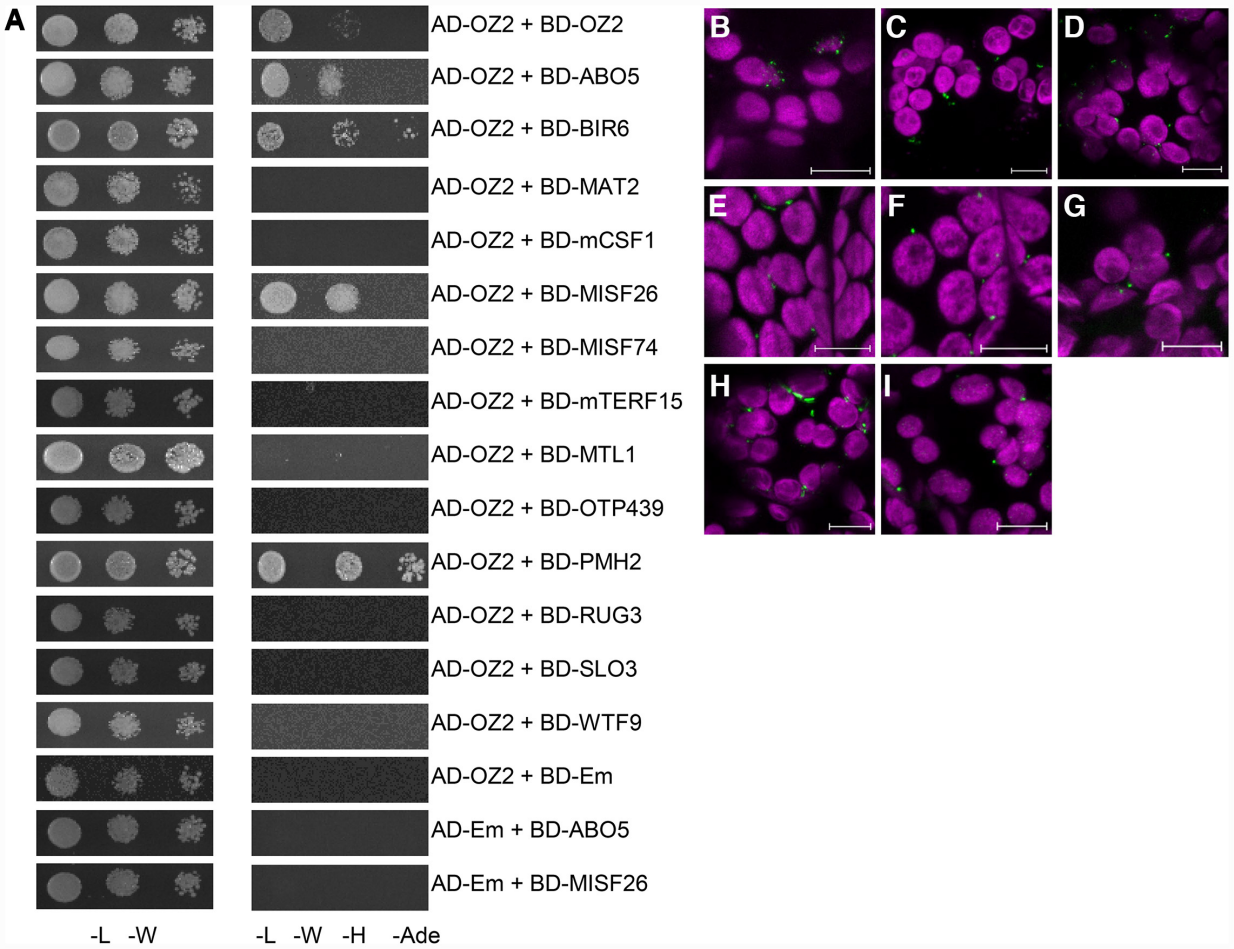
OZ2 interacts with several site-specific and general mitochondrial splicing factors *in vitro* and *in vivo*. (**A**) Yeast two-hybrid assay for OZ2/mitochondrial splice factors demonstrate interactions with certain factors that share splicing targets with OZ2, as well as OZ2 dimerization. Haploid yeast were transformed with constructs expressing OZ2 or a mitochondrial splicing factor with N-terminal fusions of parts of the yeast GAL4 transcription factor. Transformed yeast were mated to produce diploids expressing two fusion proteins and selected on synthetic dropout media lacking leucine and tryptophan (-L-W). Interactions were tested on plates lacking leucine, tryptophan, histidine, and adenine (-L-W-H-Ade). Yeast dilution spots contained 10^6^, 10^5^ and 10^4^ cells per mL. Em = empty vector. (B-I) Bimolecular fluorescence complementation (BiFC) was performed in *N. benthamiana* using transient nYFP/cYFP fusion protein expression constructs. Green = YFP fluorescence; magenta = chlorophyll autofluorescence; scale bars = 10 μm. (**B**) ABO5 + OZ2. (**C**) BIR6 + OZ2. (**D**) MAT2 + OZ2. (**E**) mCSF1 + OZ2. (**F**) MISF26 + OZ2. (**G**) MTL1 + OZ2. (**H**) PMH2 + OZ2. (**I**) OZ2 + OZ2. The interaction of OZ2 with itself serves as a positive control for this assay.

The requirement of OZ2 for certain mitochondrial splicing events may be mediated by its binding with other splicing factors. We tested the possibility of OZ2/mitochondrial splicing factor interactions by *in vitro* yeast two-hybrid (Y2H) assay and *in vivo* bimolecular fluorescence complementation (BiFC). Mitochondrial splicing factors were chosen for testing based on their sharing of target introns with OZ2; we also included factors that do not share target sites with OZ2 as possible negative controls (BIR6, MISF74 and WTF9). Some of the mitochondrial splicing factors sharing common targets with OZ2 are general factors like MAT2, mCSF1 and PMH2, which affect the splicing of several sites, while other are specific and control one to two splicing events, e.g. ABO5 and RUG3, respectively. The Y2H assays showed fewer OZ2/splice factor interactions than the BiFC assay, 4 versus 7, respectively (Figure 9, Table 1). All the interactions detected by the Y2H assay were confirmed by the BiFC assay (Table 1, Figure 9, Supplementary Figure S6). Among the four positive interactions detected by both assays, three involved splicing factors sharing targets with OZ2, one general factor (PMH2), and two specific factors (ABO5 and MISF26). One consistent interaction identified by both Y2H and BiFC assays involves BIR6, a protein that does not share a target splicing site with OZ2. The other two negative controls, MISF74 and WTF9 did not show any interaction with OZ2. The BiFC assay revealed three additional interactions not detectable by the Y2H assay, two with the general splicing factors MAT2 and mCSF1 and one with the specific factor MTL1, all of which share common splicing targets with OZ2 (Table 1, Figure 9). Overall, our interaction data are consistent with the specificity of the OZ2 splicing function being mediated in some instances by the interaction of OZ2 with other mitochondrial splicing factors.

**Table 1.** Summary of protein–protein interactions between OZ2 and mitochondrial splice factors. Y2H = yeast two-hybrid; BiFC = bimolecular fluorescence complementation

Splicing factor	Target introns	OZ2 interaction (Y2H)	OZ2 interaction (BiFC)
ABO5	nad2-i3*	Yes	Yes
BIR6	nad7-i1	Yes	Yes
MAT2	cox2, nad1-i2, nad7-i2*	No	Yes
mCSF1	rps3*, nad2-i3*, nad5-i1*, -i2*, -i3*, nad7-i2* and others	No	Yes
MISF26	nad2-i3*	Yes	Yes
MISF74	nad1-i4, nad2-i4	No	No
mTERF15	nad2-i3*	No	No
MTL1	nad7-i2*	No	Yes
OTP439	nad5-i2*	No	No
PMH2	nad5-i1*, -i2*, -i3*, rps3* and others	Yes	Yes
RUG3	nad2-i2, nad2-i3*	No	No
SLO3	nad7-i2*	No	No
WTF9	rpl2, ccmFc	No	No

*Splice sites targeted by OZ2.

## DISCUSSION

We have established the function of OZ2 in mitochondrial splicing by rescuing *oz2* homozygous mutants. These plants, in addition to a delayed growth phenotype, show a significant and pronounced reduction in the splicing of six mitochondrial introns.

**Figure 10.**
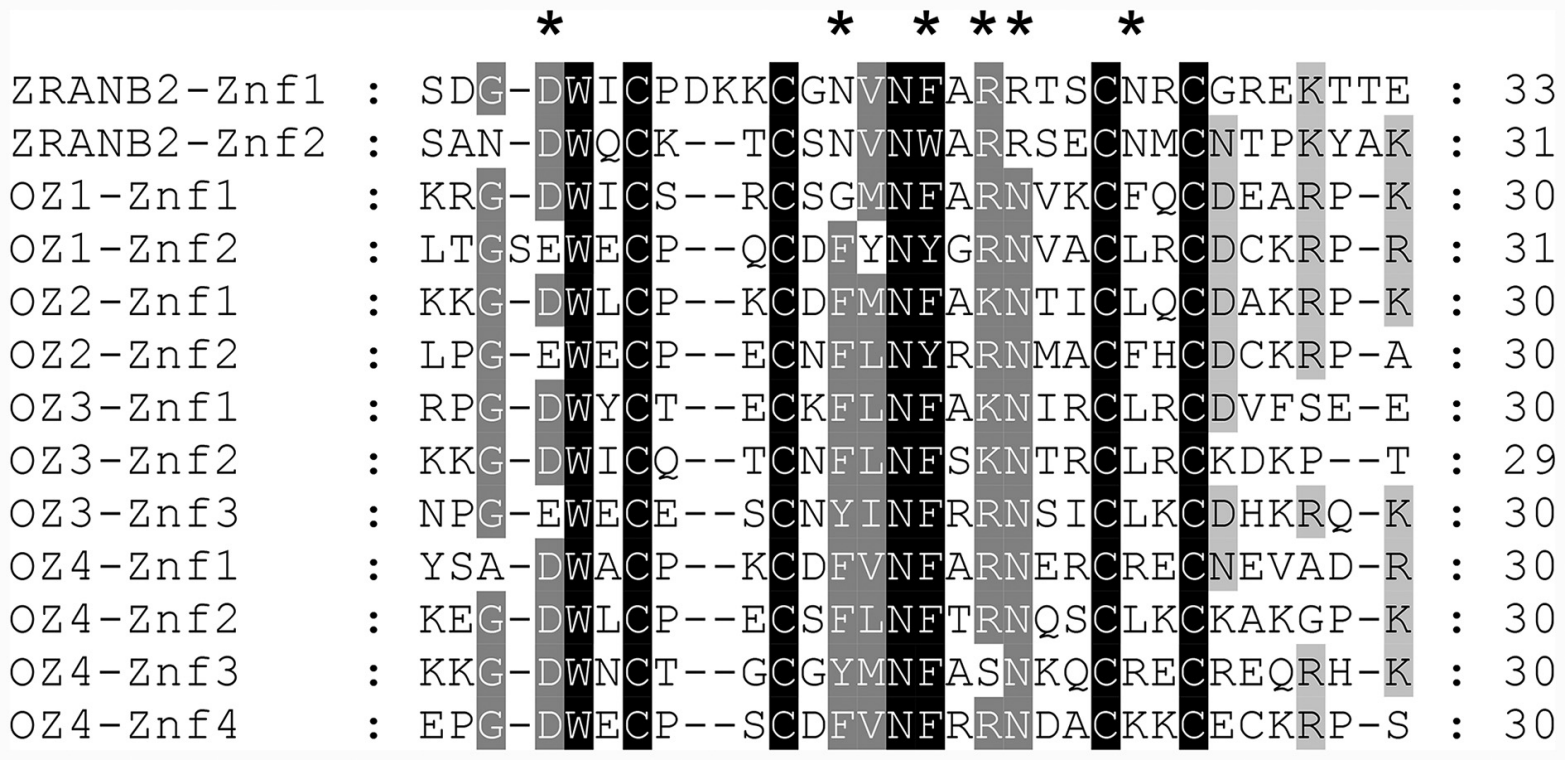
Sequence alignment of the zinc finger domains found in the human ZRANB2 protein and in Arabidopsis OZ proteins. The four conserved Cys residues distinguish this zinc finger domain from some other zinc finger families and are required for coordination of a zinc ion necessary for the structural fold of the domain. Side chain residues that directly contact RNA in ZRANB2-Znf2 are marked with an asterisk.

The only referenced domain found in OZ2 is a RanBP2-type zinc finger (PFAM00641, IPR001876), 30 residues long, which contains four cysteines required for coordinating a zinc ion that is necessary for the structural fold of the domain. Our data support the existence of a zinc ion bound to each of the zinc fingers (Znf) present in OZ2. This domain is ancient, prevalent and found in all domains of life, archaea, bacteria, and eukaryotes. Originally described in the RanBP2 nucleoporin, the domain mediates binding of RanBP2 with high specificity to exportin-1, a protein which is a key component of the nuclear export pathway (39). The RanBP2-Znf domain is found in Nup153, another nucleoporin, where it also mediates protein-protein interactions (40). More relevant to the present work is the fact that variants of the domain are found in ZRANB2, a human spliceosomal protein able to induce alternative splicing (41). The two RanBP2-type Znf domains found in ZRANB2 are able to bind ssRNA with high affinity and specificity (42). Similar Znf domains in human proteins implicated in mRNA processing are also able to bind ssRNA with a preference for a GGU motif (43). In addition to its two Znf domains located at its N-terminus, ZRANB2 contains a C-terminal arginine/serine rich (RS) domain typical of splicing factors, where it facilitates both protein-protein and protein-RNA interactions. Interestingly, OZ2 shares a similar architecture with two Znf domains, albeit in the middle of the protein, followed by a glycine-rich region in its C-terminus (*E*-value: 4.2e^−07^). The C-terminus of OZ2 is also enriched in arginine and serine, although the significance is below the threshold level (*E*-value: 0.16 and 0.35, respectively). An X-ray crystallography of the second Znf of ZRANB2 bound to a 6-nt RNA with the sequence AGGUAA was resolved at 1.4 Å and identified six residues that directly contact the RNA (42). Three of these six residues are identical or chemically similar to the ones found in the Znf domains of OZ2, suggesting that OZ2 likely binds ssRNA (Figure 10). A notable difference concerns the two asparagines that specify uridine in the GGU motif; these have been substituted with phenylalanine or leucine in OZ2 (Figure 10). Therefore, it is anticipated that, if the OZ2 Znf domains bind to RNA, they might have different specificities.

It is noteworthy that the same domain has now been implicated in both nuclear and organelle splicing. The origin of nuclear introns is still under debate; however, one theory proposes that the spliceosome and nuclear introns evolved from bacterial group II introns that invaded the eukaryotic genomes (44). Convergent evolution alone can hardly explain the numerous similarities found not only in mechanisms, two identical transesterifications resulting in lariat formation and exon junction, but also in the structures of group II and nuclear introns. In addition to the striking parallel between the function and structure of the small nuclear RNAs (snRNAs) and the group II intron RNA, some homologies are clear between Prp8, the protein core of the spliceosome, and the group II intron encoded protein (45). The discovery of OZ2 as a mitochondrial splicing factor containing a domain involved in nuclear splicing establishes another link between these two possible evolutionary related processes.

The specificity of OZ2 function in the splicing of certain introns might be mediated by interaction with other splicing factors. Eighteen splicing factors have been identified that share some common targets with OZ2; some are required for the processing of many introns like CRM-containing proteins mCSF1 and CFM9, (46,47), RNA DEAD-box helicases PMH2 and ABO6 (48,49), and maturases nMAT1, nMAT2, nMAT4 and MatR (10,50–52). Other more specific splicing factors are involved in the removal of one to two introns; most of these proteins belong to the PPR family (OTP43, MSP1, ABO5, MISF26, OTP439, TANG2, SLO3, MTL1) (22,34,37,53–56). mTERF15 and RUG3 are the only representatives of their families that have been shown to be required for the splicing of mitochondrial introns (*nad2*-intron 3, and *nad2*-intron 2 and intron 3, respectively) (57,58). We chose to test the interactions of OZ2 with ten of these factors, both the specific and the general factors, and included three putative negative controls represented by splicing factors that do not share targets with OZ2. Our results clearly indicate that OZ2 can interact not only with general splicing factors like PMH2, mCSF1 and MAT2, but also with more specific factors such as ABO5 and MISF26 (Table 1). Interestingly, these two factors are involved in the splicing of the same intron, the third intron in the *nad2* transcript. Many chloroplast splicing factors have been found in high molecular weight ribonucleoprotein complexes by sucrose gradient sedimentation experiments (59–63). Similar experiments have shown that PMH2 and nMAT2 are also associated with their intron-RNA targets in large ribonucleoprotein particles *in vivo* (64). Our results suggest that OZ2 might also be found in these high molecular weight ribonucleoprotein complexes, particularly the one controlling the splicing of the third intron in the *nad2* transcript. The localization of OZ2 as punctate foci in Arabidopsis mitochondria as seen in Figure 2A might be caused by its interaction with other splicing factors.

An inconsistency in our interaction data concerns BIR6, a protein shown to interact with OZ2 by both Y2H and BiFC assays even though it does not share a common splicing target with OZ2. BIR6 is involved in the splicing of intron 1 of the mitochondrial *nad7* transcript (21). However, two other splicing events are affected in the *bir6* mutants though to a much lesser extent than the splicing of *nad7*-intron 1, the removal of introns 1 and 2 from the *nad2* transcript (Figure 4A in (21)). The significance of the effect of OZ2 on the splicing of *nad2*-intron 1 is just above the threshold level (*P* = 0.051). The interaction between BIR6 and OZ2 might thus be explained by their minor involvement in the splicing of the first intron of the *nad2* transcript.

The BiFC assay revealed three additional interactions when compared to the Y2H assay. The heterologous system represented by the Y2H assay might not allow the proper folding of some of these factors preventing their interactions. In addition, we removed the transit peptide from the factors assayed in the Y2H assay based on software predictions; a possible error in the predicted cleavage sites might have led to the removal of too much or too little of the protein, impairing its proper folding.

Splicing efficiency is a metric often used to measure the effect of a splicing factor and is calculated as the ratio of spliced to unspliced forms of each transcript in the mutant normalized to the same ratio in the wild-type plant; it is generally expressed as a base 2 logarithm ratio. The reduction in splicing efficiency in mutants affected either in general factors such as CFM9 or nMAT1 or in factors having another function such as ODB1 or MTL1 is moderate and ranges from 2^−1^ to 2^−6^ (34,47,50,65). On the other hand, mutants in specific factors like PPR proteins tend to exhibit a more pronounced decrease in splicing efficiency, ranging from 2^−6^ to 2^−28^, as observed for SLO3 and OTP43, respectively (22,56). *oz2* mutant plants exhibit a marked decrease in splicing efficiency, varying between 2^−6^ (*nad2*-intron 3, *nad5*-intron 1) and 2^−10^ (*nad7*-intron 2). Furthermore, the rescued *oz2* mutant plants are not null mutants with a complete absence of OZ2, as the ABI3 promoter is leaky and allows a residual expression of the transgene under its control. Therefore, the effect of OZ2 in its scope is more comparable to specific splicing factors even though it affects more targets. This specific effect is also reflected by the number of defective splicing events, one to two, observed per transcript. For instance, the splicing efficiency of *nad1*-intron 1 is reduced to 2^−8^ in *oz2*, while the other introns in *nad1* transcript are not affected (Figure 3B).

Whatever the molecular mechanism by which OZ2 is involved in the splicing of mitochondrial introns, its impairment leads to a reduction in complex I assembly and activity and a subsequent morphological phenotype characteristic of complex I mutant plants (Figure 6). All the other mitochondrial complexes were similar in abundance and activity between the *oz2* mutant and the wild-type plants. Complex I mutant plants have been studied in different species and originate from: 1) deletion in mitochondrial genomes, e.g. the CMSI (cytoplasmic male sterile) and CMSII plants in *Nicotiana sylvestris* (66), the NCS2 (non-chromosomal stripe) mutant in maize, 2) point or T-DNA insertional mutation in nuclear genes encoding mitochondrial subunits like the *fro1* (frostbite1), *ndufs4* or *ca2* mutants in Arabidopsis (67–69), and 3) nuclear mutants in genes encoding factors required for the maturation of mitochondrial transcripts, like NMS1 plants in *Nicotiana sylvestris* (70) and the *otp43* and *mtsf2* mutant plants in Arabidopsis (22,35). The range of phenotypes observed in these mutants varies quite dramatically both between and within species regarding embryo development and vegetative growth. In NCS2 maize mutant plants, deletion of the 3′ end of the *nad4* gene causes lethality during kernel development; only heteroplasmic plants that contain normal mitochondria in addition to mutant ones can be propagated. The segregation of mutant mitochondria during development results in mutant sectors that appear as pale stripes, hence the name of the mutants (71). The *Nicotiana sylvestris* CMS plants exhibit delayed development and conditional male sterility depending on light intensity (66). In Arabidopsis, the *ca2* mutants affected in a gene encoding a plant specific subunit of complex I with similarity to carbonic anhydrases do not show any morphological phenotype even though Complex I activity was decreased by 80% (69). On the other hand, Arabidopsis *otp43* mutant plants defective in the *trans*-splicing of *nad1*-intron 1 exhibit a total lack of complex I activity; in addition to delayed development and severe morphological phenotype, the mutant seeds require germination on half-strength MS medium supplemented with 1% sucrose (22). This mutation belongs to the comprehensive dataset of 510 cloned *EMBRYO-DEFECTIVE* (*EMB*) genes established by Meinke (13). Thus, it appears that the effect of a complex I dysfunction on embryo development in Arabidopsis depends on the severity of the reduction in the activity of this complex. Measurement by in-gel activity assay lacks the sensitivity to detect trace amounts of complex I activity. This limitation might explain the ambiguity surrounding the essential function of complex I in embryo development in Arabidopsis. For instance, *ndufs4* mutants lacking the 18-kDa subunit of complex I that do not need sucrose to germinate were first reported to lack complex I activity (68), but in a later report were found to exhibit trace amounts of activity (72). A recent report on *msp1* mutant plants similarly affected in *trans*-splicing of *nad1*-intron 1 as *otp43* mutant plants further supports the essential role of complex I in embryo development (53). Like *otp43*, the *msp1* mutation is annotated as an EMB gene (EMB1025) (13). A modified method for embryo rescue of *msp1* plants had to be developed by sowing mutant seeds on MS-agar plates supplemented with 1–3% sucrose and various vitamins (53).

The *oz2* mutation was first reported as the *slow embryo development1* (*sed1*) mutation, which results in retarded embryogenesis and aborted seeds (12). Light microscopy data indicated that embryonic arrest occurs by approximately early globular stage in *sed1* (*oz2*) seeds. Attempts to rescue the mutant embryos by growing them on half-strength MS medium failed as they died after 3 weeks (12). What can be the origin of the embryo defect caused by the *oz2* mutation? Given the splicing deficiency in *rps3* (the gene encoding the mitochondrial ribosomal protein S3) and *nad* transcripts observed in *oz2* rescued mutants, both a defective mitochondrial translation apparatus and/or complex I could explain the embryo phenotype. NCS3 and NCS4 maize mutants present deletions of the mitochondrial *rps3* and are associated with very reduced levels of mitochondrial protein synthesis (73,74). Surprisingly, the abundance of Complexes III, IV and V was not reduced in *oz2* rescued mutant plants (Figure 6 and Supplementary Figure S5) even though they contain mitochondrial-encoded subunits, suggesting that the reduced *rps3* mature transcript does not impair mitochondrial protein synthesis. However, as noted earlier, expression under the control of the ABI3 promoter is not totally turned off during seedling development, the stage at which the RNA was extracted from *oz2* rescued plants. It is possible that the amount of *rps3* spliced product is more drastically reduced during the embryo development of *oz2* mutant, thus contributing to its arrest. The cumulative effect of splicing deficiencies in several *nad* transcripts probably explains the observation of a reduced complex I abundance and activity in *oz2* rescued mutant plants. Like for *rps3*, the decrease in *nad1, 2, 5* and *7* mature transcripts, and hence in complex I, is likely to be more severe in an *oz2* mutant that has not been rescued by the ABI3 promoter.

Like many mutants affected in complex I biogenesis, *oz2* rescued mutants show an induction of alternative respiratory pathway genes, especially *AOX1d* whose transcript steady state level was ∼270-fold higher than in the wild-type plants (Figure 7). This observation is reminiscent of the strong induction of AOX1d at the transcript and protein level in a double mutant impaired in both the cytochrome c oxidase pathway and the AOX pathway (*aox1a:rpoTmp*) (75). The induction of AOX1d could not compensate for the absence of AOX1a, as the double mutant exhibited a more severe growth retardation than the single *rpoTmp* mutant suggesting differential activity of AOX isoforms. A differential activity of AOX isoforms was later substantiated by a differential response to tricarboxylic acid cycle intermediates (76). Another feature shared by *oz2* rescued mutant plants and complex I mutants is the upregulation of steady state levels of mitochondrial transcripts, which may correspond to compensatory effects due to the altered organellar functions (Supplementary Figure S3), e.g. (50,51,54,58).

In this work, we have investigated the function of OZ2 and discovered its involvement in mitochondrial splicing. Another OZ family member, OZ1, was previously identified to be a chloroplast editing factor (77). The remaining members of this family, OZ3 and OZ4, both predicted to be localized either in the mitochondrion or in the plastid (30) have not yet been assigned any function, though it seems likely that they will be found to have a role in organelle RNA metabolism.

## Supplementary Material

gkab066_Supplemental_File

## Notes

Present address: Alexander Kehl, Biophysics Graduate Group, University of California, Davis, CA 95616, USA.

Present address: Lauren Hamm, Plant and Microbial Biology, University of California, Berkeley, CA 94703, USA.
