# Supplementary material for: A RanBP2-type zinc finger protein functions in intron splicing in Arabidopsis mitochondria and is involved in the biogenesis of respiratory complex I
Source: Nucleic Acids Res. 2021 Feb 28;49(6):3490–506. doi: 10.1093/nar/gkab066 (PMC8034646; doi:10.1093/nar/gkab066)
Supplement: gkab066_Supplemental_File [file gkab066_supplemental_file.pdf]

Name	sequence	purpose
SALK_043472C-LP	AGATGTGCCTAGCTTCTGCTG	genotyping oz2 segregating progeny
SALK_043472C-RP	AGCTTAGGGCTTTGAAATTCG	
LBb1.3	ATTTTGCCGATTTCGGAAC	
CS84933-LP	ATTCTCCTGGAAGGAGCTGTC	genotyping oz2 segregating progeny
CS84933-RP	ACTCCCTCTTGAGAATCCTGG	
p745	AACGTCCGCAATGTGTTATTAAGTTGTC	
ABI3-F2	CTCAAAACGTAAAACAACGTCATT	sequencing the ABI3 promoter
ABI3-F3	AATCCGTGTCTGCCTCTTCC	
ABI3-F4	CTCTCCTTTTCCTTCTGCTGAG	
ABI3-F5	GCTTTGGATCCTCCTTCGTC	
OZ2-F1	ATGGCTGCTTCAATCTCTCTTCTTCTC	cloning of OZ2 CDS
OZ2-R1	CTATCTCTCGATAACTCTTCGACTGTTTCTG	
OZ2-F2	CAGATTGATTCTGGTGAGTTGAGTA	sequencing the OZ2 CDS
OZ2-F3	GTCTCCTGAAGAAAAAGTCTGTTAA	
OZ2-F4	ATTCCAGTAAAAAGAATGAAAACCT	
OZ2-F5	TCGATTCTGATATGGAGGATGATAG	
AtOZ2_ZnFing_For	CTGTGAATTCCCAGTTATCAAAAGAGCGCCC	expression of the recombinant protein used in zinc binding analysis
AtOZ2_ZnFing_Rev	CTGTAAGCTTTTACCTGTCTGTTTGTGCTTCAGTG	
OZ2-nostop-R1	TCTCTCGATAACTCTTCGACTGTTTCTG	OZ2 localization and BiFC
qRTnad2-ex2-F	ATTGAGCCTCAAAGTTTATGTTTTTATGT	quantitative RT-PCR for nad2-ex2ex3 (spliced): qRTnad2-ex2F+qRTnad2-ex3R
qRTnad2-ex3R	ATCGAGCACCAGTGATTTCGTATCC	
qRTnad2-in2R	GATATCGGTAGTTGTCCGGTCGTACC	quantitative RT-PCR for nad2-ex2in2 (unspliced): qRTnad2-ex2F + qRTnad2-in2R
qRT-AOX1b-F	AGAAAACTACGGAGGAGAAAGG	quantitative RT-PCR for AOX1b
qRT-AOX1b-R	ATATGTCTCCCATGGCCTAAAG	
qRT-AOX2-F	TATCGACAATGGGAAGATCGAG	quantitative RT-PCR for AOX2
qRT-AOX2-R	CCGTTACAACATCTTTCAACGT	
PPE-nad2-ex3ex4	/5HEX/CCCTCATAGATATCTGGTGCCCACATAT	primer used in the PPE reaction to determine the splicing of nad2-intron3
PPE-nad7-ex2ex3	/5HEX/CGTGTTCTTGGGCCATCATAGAAACATAG	primer used in the PPE reaction to determine the splicing of nad7-intron2
OZ2-Y2H-F	AGCAACACTGAATCAACCCATGAA	primers used to amplify genes whose products were tested in Y2H
ABO5-Y2H-F	GCCACAAAGTATGTCGCCAAAG	
ABO5-Y2H-R	CTACAAAGGGCTGACAACCCA	
BIR6-Y2H-F	TCTTCGAAACCAGATTCTATGCTT	
BIR6-Y2H-R	TCAAGCTGCAGCACCAAAG	
MAT2-Y2H-F	TGGCTAAAACCTTCTTCTACATATACC	
MAT2-Y2H-R	TTACATGCGTGCAATGCGAAGC	
mCSF1-Y2H-F	GCCTCCGAAAATCCTGAC	
mCSF1-Y2H-R	CTAGGTTGTCTCGTCAGGAG	
MISF26-Y2H-F	GCATTGACGATTCATATCCTTGTCAAAGC	
MISF26-Y2H-R	TCATCTGACTGAAATCATCTCATTATAAACTCTATC	
MISF74-Y2H-F	GAAGCTCGAAAACCGATTGTCTCG	
MISF74-Y2H-R	CTATAAATGTCTCCTCCTCTCTCTAGCA	
mTERF15-Y2H-F	TCCTCACGAATTCTCACACCTATCAA	
mTERF15-Y2H-R	CTAAGCAAGTGACTCTATAAAGGAC	
MTL1-Y2H-F	TCTTTGCAAAGAATCTGCTACTACG	
MTL1-Y2H-R	TCAAAAAGCTGCATTATAAAACCTTCG	
OTP439-Y2H-F	AATCTCAATGTGAATCATCTCCTC	
OTP439-Y2H-R	CTATATGGTAAACTGATCATGGG	
PMH2-Y2H-F	GCTGGATTTGCGATCTCTG	
PMH2-Y2H-R	TCAGTAAGATCTTTTCCCATCATTTG	
RUG3-Y2H-F	ACAAGCCCAGACATCGACTCCG	
RUG3-Y2H-R	TTAAGGTGATCTTGAGACTAAACACAGAGC	
SLO3-Y2H-F	TCTTCATCTTTATCATCTACATCGA	
SLO3-Y2H-R	CTACATTAGCTGTATTTCAGGAGGAG	
WTF9-Y2H-F	CACTTCTTAAGGAAGTTTCCTTCAATCTTTG	
WTF9-Y2H-R	TTAGCCTTCAAAATCCAAATCCAAATCTTTATC	
ABO5-BiFC-F	ATGAAGCTTCTCCGCCGC	primers used to amplify genes whose products were tested in BiFC
ABO5-BiFC-R	CAAAGGGCTGACAACCCAC	
BIR6-BiFC-F	ATGTACAGATCAATGGCAATCCTG	
BIR6-BiFC-R	AGCTGCAGCACCAAAGAGTTC	
MAT2-BiFC-F	ATGCGTAGAAGCTTCTCTGTTTTGGG	
MAT2-BiFC-R	CATGCGTGCAATGCGAAGC	
mCSF1-BiFC-F	ATGTTCTTGATTCGTCTCTCCCG	
mCSF1-BiFC-R	GGTTGTCTCGTCAGGAGAATCTTG	
MISF26-BiFC-F	ATGGCGTCAGCTTTGCG	
MISF26-BiFC-R	TCTGACTGAAATCATCTCATTATAAACTCTATCAGC	
MISF74-BiFC-F	ATGATTCGCCGGCCGATC	
MISF74-BiFC-R	TAAATGTCTCCTCCTCTCTCTAGCATTTTTACG	
mTERF15-BiFC-F	ATGGCCTCAAAACTCAAAACCTTCA	
mTERF15-BiFC-R	AGCAAGTGACTCTATAAAGGACTTCATGT	
MTL1-BiFC-F	ATGGTTATGCTAGCGAGATCAAAGCTAG	
MTL1-BiFC-R	AAAAGCTGCATTATAAAACCTTCGTCTAT	
OTP439-BiFC-F	ATGTACTTGAAAGAAGGATGTTCG	
OTP439-BiFC-R	TATGGTAAACTGATCATGGGCTCT	
PMH2-BiFC-F	ATGATCACTACAGTGCTACGACGA	
PMH2-BiFC-R	GTAAGATCTTTTCCCATCATTTGA	
RUG3-BiFC-F	ATGGCAGCGTTAAGCCACC	
RUG3-BiFC-R	AGGTGATCTTGAGACTAAACACAGAGC	
SLO3-BiFC-F	ATGCTTCAGAAGATCTCCTCCGAT	
SLO3-BiFC-R	CATTAGCTGTATTTCAGGAGGAGG	
WTF9-BiFC-F	ATGCTCTCTATTCGCCGCCA	
WTF9-BiFC-R	GCCTTCAAAATCCAAATCCAAATCTTTATCTAC	

Supplementary Table S1. List of primers used in this study

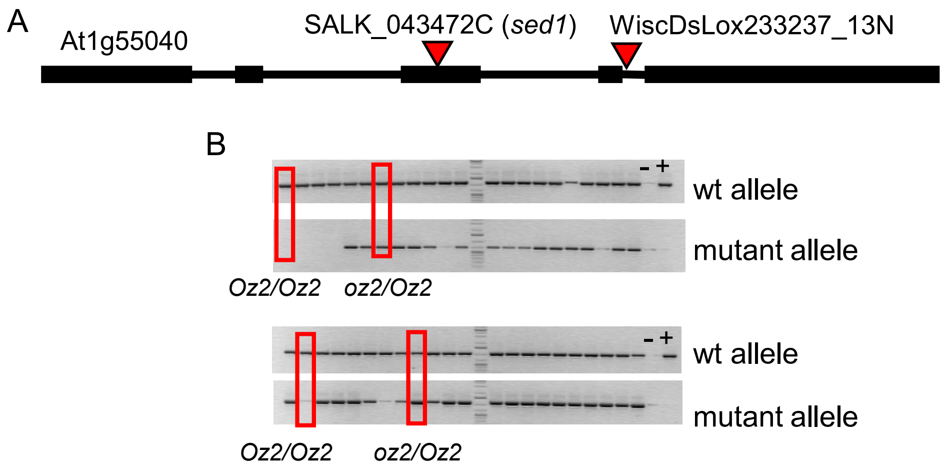

Supplementary Figure S1. oz2 mutation is embryo lethal. A. Gene model for OZ2 (At1g55040). Exon (introns) are represented by black squares (lines). Two insertional T-DNA mutant lines are available, and the location of the insertion is represented by a red triangle. B. Genotyping progeny of oz2/OZ2 from two independent insertional mutations shows an absence of homozygous mutant plants oz2/oz2. Upper panel progeny from SALK_043472C, lower panel progeny from WiscDsLox233237_13N. Only homozygous wild-type (Oz2/Oz2) or heterozygous (oz2/Oz2) plants are present.

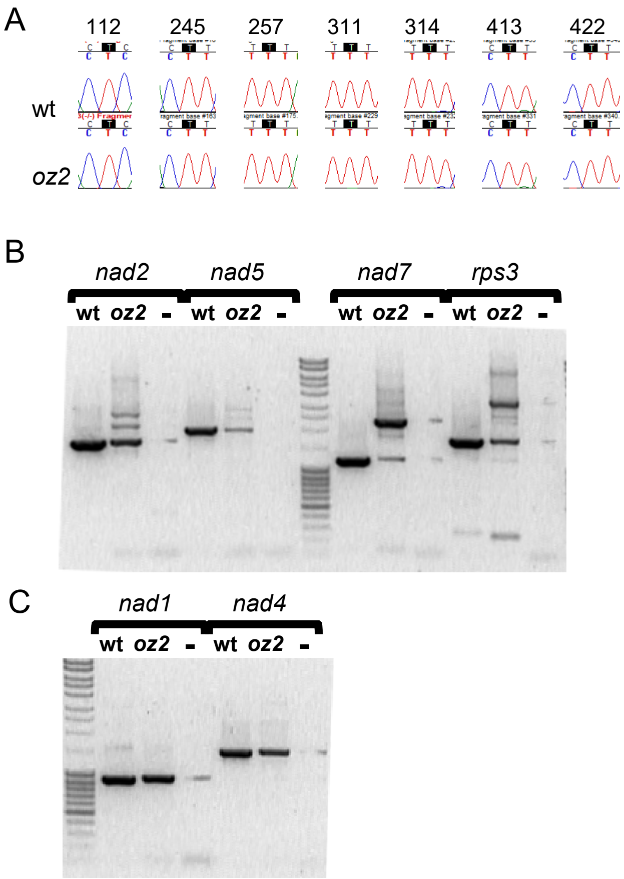

Supplementary Figure S2. OZ2 is not an editing factor. A. Example of the editing survey of *oz2* mutant performed by bulk sequencing of RT-PCR products. These electrophoretograms were obtained by sequencing the mitochondrial gene *cox3* from a wild-type (wt) plant and from an *oz2* homozygous mutant plant. Above each electrophoretogram is the position of the edited cytidine in the *cox3* transcript. Each cytidine is fully edited (only a T peak) in both wt and *oz2*. This survey was repeated for the whole set of mitochondrial genes known to harbor editing sites. Only spliced products containing only exons were analyzed. Intron sequences were not included in the analysis. B. RT-PCR products for *nad2*, *nad5*, *nad7* and *rps3* show a ladder pattern in the *oz2* mutant plant with a marked reduction of the mature product. C. RT-PCR products for *nad1* and *nad4* show a slight reduction of the mature product in the *oz2* mutant.

Target	P-Value ANOVA	P-Value BH	Ratio *oz2*/wt
atp1	3.9E-03	2.4E-02	5.5E+00
ccmFc-ex1ex2	5.7E-03	3.2E-02	9.5E+00
ccmFn2	2.9E-03	2.2E-02	3.8E+00
cox1	1.2E-02	4.8E-02	3.4E+00
matR	2.7E-03	2.2E-02	3.3E+00
nad1-ex2ex3	3.5E-03	2.4E-02	6.9E+00
nad1-ex3ex4	6.6E-03	3.2E-02	4.8E+00
nad1-ex4ex5	6.1E-03	3.2E-02	5.1E+00
nad1-in1ex2	9.8E-03	4.1E-02	4.5E+00
nad1-in4ex5	1.5E-03	1.6E-02	6.1E+00
rps3-ex1in1	1.7E-03	1.6E-02	9.7E+00
nad1-ex1ex2	9.4E-07	6.4E-05	1.7E-02
nad2-ex3ex4	5.5E-04	9.3E-03	1.2E-03
nad5-ex1ex2	6.8E-04	9.3E-03	4.3E-02
nad5-ex2ex4	5.8E-04	9.3E-03	1.7E-02
nad7-ex2ex3	6.4E-04	9.3E-03	1.1E-03
rps3-ex	8.9E-03	4.0E-02	1.1E-01

Supplementary Figure S3. Transcript species showing a significant difference between oz2 mutant plants and wild-type plants. Figure S3 gives the results from the one-way analysis of variance (ANOVA) between the wild-type group (three plants) and the mutant *oz2* plant group (three T1 and one T0). In red (blue) background the amount of the transcript species is significantly increased (decreased) in the oz2 mutant plant. The increase ranges from 3.3 (matR) to 9.7 (rps3 unspliced) while the decrease is much more pronounced and ranges from 0.11 (rps3 spliced) to 0.001 (nad7-ex2ex3). The decrease in transcript amount in the oz2 mutant affects only spliced species. Among 68 transcript species tested by qRT-PCR, 11 exhibit an increase in the oz2 mutant plant (red background), while 6 show a decrease (blue background). The P-Value ANOVA is not corrected for multiple testing, while the P-value after Benjamini-Hochberg (BH) is corrected for false discovery rate.

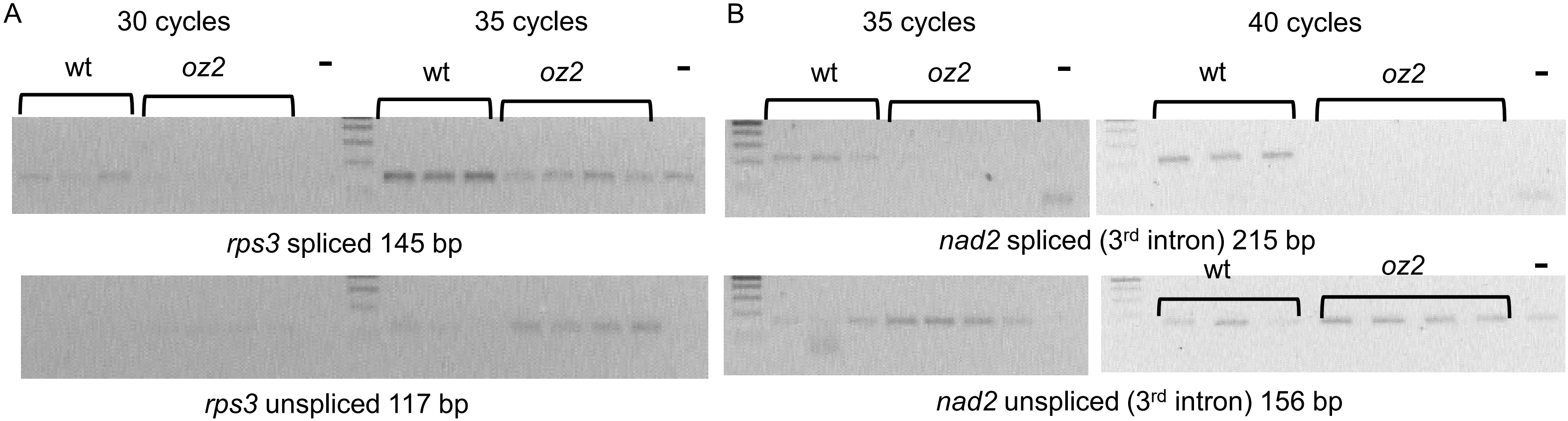

Supplementary Figure S4. Semi-quantitative RT-PCR assay confirms that *oz2* mutation results in a splicing defect. The amount of template for each PCR reaction was the same (1 ng) and estimated after quantifying the RNA for each wild-type and oz2 mutant plant by Qubit assay. A. *rps3* assay shows a detectable amount of spliced product in the wt at 30 cycles, while there is no detectable product in the oz2 lanes. Conversely some unspliced product is detectable in the oz2 lanes at 30 cycles while no product is apparent in the wt lanes. At 35 cycles, some spliced product is detectable in the *oz2* lanes while the amount of amplified spliced product is higher in the wt lanes (more intense bands). A similar observation can be made for the unspliced products which is now detectable in wt lanes, while more abundant in the *oz2* lanes. B. Semi-quantitative RT-PCR assay for the splicing of the *nad2* third intron. At 35 cycles, some spliced product is detectable in the wt lanes, but none in the *oz2* lanes. The unspliced product is detectable in all the lanes but is more abundant in the *oz2* lanes. At 40 cycles, the spliced product is readily visible in the wt lanes while still not detectable in the *oz2* lanes. As at 35 cycles. the amount of unspliced product is more abundant in the *oz2* lanes than in the wt lanes.

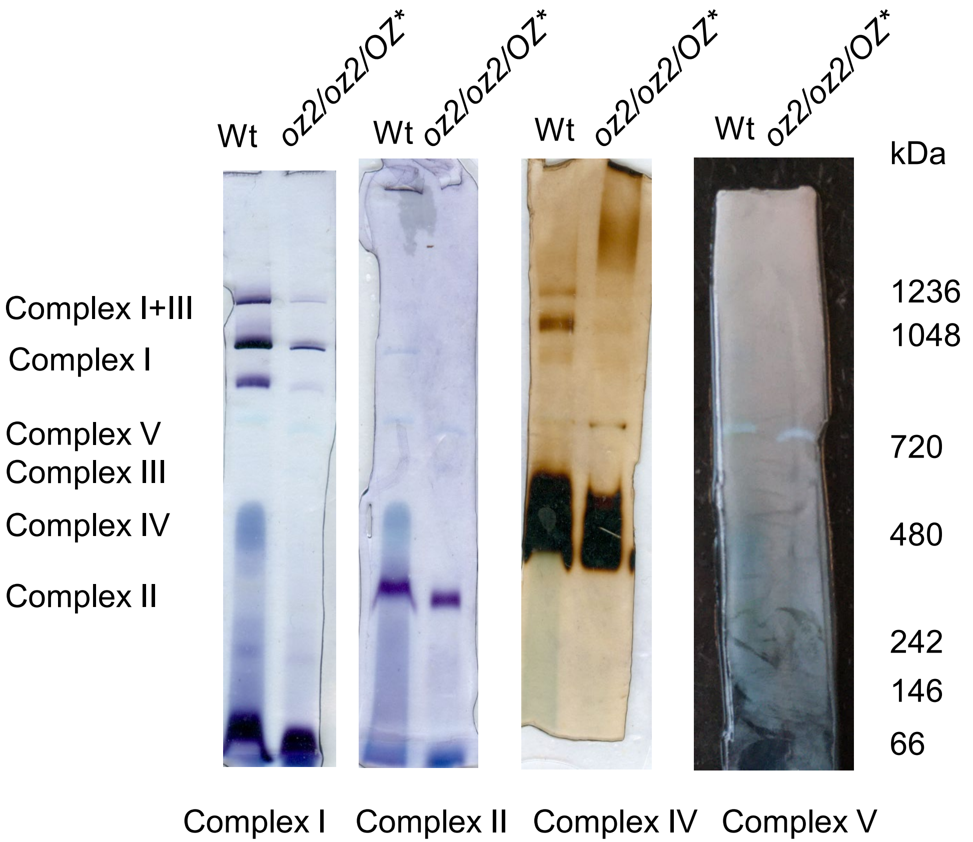

Supplementary Figure S5. In-gel activity assays of mitochondrial enzymes in native gels demonstrate that only complex I is seriously impaired in the oz2/oz2/OZ2* mutant. Arabidopsis mitochondrial proteins were solubilized by digitonin and subsequently separated by 1-D BN-PAGE. After electrophoresis, slices of the gel were stained with in gel activity assays for complex I, complex II, complex IV, or complex V.

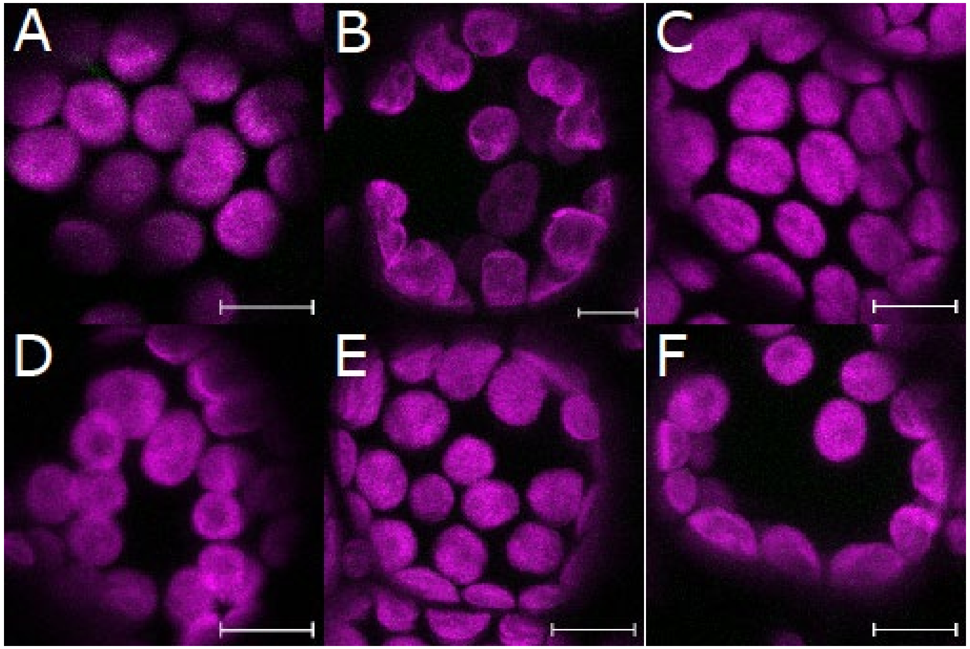

Supplementary Figure S6. Negative results from OZ2 BiFC performed in *N. benthamiana* using transient nYFP/cYFP fusion protein expression constructs. Green = YFP fluorescence; magenta = chlorophyll autofluorescence; scale bars = 10 µm. (**A**) MISF74 + OZ2. (**B**) mTERF15 + OZ2. (**C**) OTP439 + OZ2. (**D**) RUG3 + OZ2. (**E**) SLO3 + OZ2. (**F**) WTF9 + OZ2.
